# Investigation and Functional Characterization of Rare Genetic Variants in the Adipose Triglyceride Lipase in a Large Healthy Working Population

**DOI:** 10.1371/journal.pgen.1001239

**Published:** 2010-12-09

**Authors:** Stefan Coassin, Martina Schweiger, Anita Kloss-Brandstätter, Claudia Lamina, Margot Haun, Gertraud Erhart, Bernhard Paulweber, Yusof Rahman, Simon Olpin, Heimo Wolinski, Irina Cornaciu, Rudolf Zechner, Robert Zimmermann, Florian Kronenberg

**Affiliations:** 1 Division of Genetic Epidemiology, Department of Medical Genetics, Molecular and Clinical Pharmacology, Innsbruck Medical University, Innsbruck, Austria; 2 Institute of Molecular Biosciences, University of Graz, Graz, Austria; 3 First Department of Internal Medicine, Paracelsus Private Medical University Salzburg, Salzburg, Austria; 4 Department of Inherited Metabolic Diseases, Evelina Children Hospital, Guys and St. Thomas's National Health Service Foundation Trust, London, United Kingdom; 5 Department of Clinical Chemistry, Sheffield Children's Hospital, Sheffield, United Kingdom; 6 Structural Biology Group, Institute of Molecular Biosciences, University of Graz, Graz, Austria; University of Alabama at Birmingham, United States of America

## Abstract

Recent studies demonstrated a strong influence of rare genetic variants on several lipid-related traits. However, their impact on free fatty acid (FFA) plasma concentrations, as well as the role of rare variants in a general population, has not yet been thoroughly addressed. The adipose triglyceride lipase (ATGL) is encoded by the *PNPLA2* gene and catalyzes the rate-limiting step of lipolysis. It represents a prominent candidate gene affecting FFA concentrations. We therefore screened the full genomic region of *ATGL* for mutations in 1,473 randomly selected individuals from the SAPHIR (Salzburg Atherosclerosis Prevention program in subjects at High Individual Risk) Study using a combined Ecotilling and sequencing approach and functionally investigated all detected protein variants by in-vitro studies. We observed 55 novel mostly rare genetic variants in this general population sample. Biochemical evaluation of all non-synonymous variants demonstrated the presence of several mutated but mostly still functional ATGL alleles with largely varying residual lipolytic activity. About one-quarter (3 out of 13) of the investigated variants presented a marked decrease or total loss of catalytic function. Genetic association studies using both continuous and dichotomous approaches showed a shift towards lower plasma FFA concentrations for rare variant carriers and an accumulation of variants in the lower 10%-quantile of the FFA distribution. However, the generally rather small effects suggest either only a secondary role of rare *ATGL* variants on the FFA levels in the SAPHIR population or a recessive action of *ATGL* variants. In contrast to these rather small effects, we describe here also the first patient with “neutral lipid storage disease with myopathy” (NLSDM) with a point mutation in the catalytic dyad, but otherwise intact protein.

## Author Summary

The nature of the genetic variation underlying common traits is not yet completely understood. Recently, there has been a shift in the genetic research focus towards the elucidation of the influence of rare variants, which are thought to exert a strong impact on common traits. Circulating free fatty acids are immediate products of the triglyceride breakdown and represent a yet poorly addressed phenotype to study the impact of rare variants on lipolysis. Since ATGL (encoded by the *PNPLA2* gene) controls the rate limiting step of lipolysis, we screened its whole gene region in 1,473 healthy individuals and found that 1 out of 13 individuals indeed carried at least one rare mutation. Biochemical investigations showed that, even in a healthy population, several missense variants lead to an impaired catalytic activity and 1 variant even produced a completely inactive protein. However, subsequent association studies revealed only small effects on the free fatty acids levels in the population. This suggests an only secondary role of rare ATGL variants on free fatty acids levels. More generally, we conclude that even in a healthy population pronounced allelic heterogeneity due to the presence of several rare variants may be common.

## Introduction

Despite immense efforts, the genetic contribution to many complex phenotypes and diseases is still incompletely understood [1]. Two opposite hypotheses about the nature of genetic variation underlying common diseases have been proposed. The “common disease – common variant” hypothesis argues that the genetic basis of complex diseases is formed by the cumulative action of frequent polymorphisms (here defined by a minor allele frequency (MAF) >5%) with small impact [2], while the “common disease – rare variant” hypothesis rather advocates a strong impact of rare variants with large effects and high penetrance [3], [4]. Genome-wide association studies (GWAS) based on the former rationale led to the identification of an impressive wealth of new loci and pathways [5], [6], but left a large fraction of the estimated heritability still unexplained [7]. Since effects of rare variants are not captured by conventional GWAS, they were discussed to be a major source to explain this “missing heritability” [1], [7]–[9].

Studies resequencing the coding regions of candidate genes for example for lipoprotein metabolism [10]–[13], obesity [14], renal function [15], type 1 diabetes [16] and cancer predisposition [17]–[19] follow the rationale that mutations with large effects are likely to be enriched in the extremes of the phenotype distribution [8], [10]. These studies detected several rare mutations and underscored the power of the “extreme phenotype-approach” to identify new functional variants and provide substantial insights into the molecular function of genes [20]–[22]. However, the influence of rare variants outside of phenotypic extremes and their relevance in the general population has not yet been addressed in depth. Some expectations about the effect size of rare variants may currently be too optimistic and rare variants with modest but still detectable effects are likely to exist [23]. In addition, resequencing studies so far focused on coding regions, neglecting promoter regions and introns. Finally, recently resequenced genomes revealed that current single nucleotide polymorphism (SNP) databases are still far from being complete [24]–[29].

**Figure 1:**
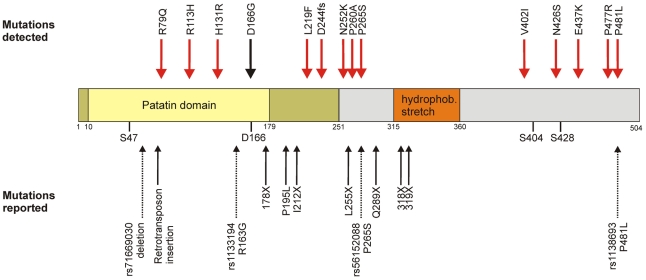
Domain organization and genetic variability of the ATGL protein. Upper panel: Amino acid exchanges found in SAPHIR (red) and the patient with neutral lipid storage disease with myopathy (black). Central panel: Graphical representation of the *ATGL* domain organization as described in the main text. Dark yellow: α/β hydrolase fold. Yellow: Patatin domain with catalytic residues S47 and D166. Orange: hydrophobic stretch. Grey: C-Terminus. S404 and S428: phosphorylated serine residues. Lower panel: Already known variations. Bold arrows: Mutations known to cause NLSDM [41], [46]. Dotted arrows: known SNPs from dbSNP b.131.

Rare genetic variants at a population level have not been investigated for *PNPLA2* (more commonly known as Adipose Triglyceride Lipase, *ATGL* [MIM *609059]) which is a highly interesting candidate gene in the lipolytic cascade. The protein encoded by the *ATGL/PNPLA2* gene catalyzes the rate-limiting step of lipolysis, i.e. the breakdown of triacylglycerol to diacylglycerol [30]–[32], and represents the main lipase for basal and hormone stimulated lipolysis [33]. The human ATGL protein consists of 504 amino acids divided into an N-terminal part containing the catalytic patatin domain (amino acids 10 to 178) and a more loosely defined regulatory C-terminus [34] (Figure 1). The predicted catalytic dyad is formed by a serine residue at position 47 and an aspartate residue at position 166 [33]. Moreover, a putative hydrophobic lipid binding domain at positions 315–360 and two phosphorylation sites with unknown function at Ser404 and Ser428 were described [30], [35].

Two proteins are known to regulate ATGL activity in an opposite manner. Interaction with ABHD5 (also known as CGI-58, [MIM *604780]) enhances the catalytic activity up to 20-fold in mice and still 4-fold in humans [36], while the G0/G1 switch 2 protein (*G0S2*; [no MIM number assigned]) inhibits ATGL activity by interacting with the patatin domain, as described very recently by Yang and colleagues [37]. Additionally, regulation by nutritional status, insulin, *PPARA* (MIM +170998) and *PPARG* (MIM *601487) was reported [38], [39].

Mutations in *ATGL* and *ABHD5* have provided the molecular basis underlying neutral lipid storage disease (NLSD) in humans. NLSD is a group of rare, autosomal recessive disorders characterized by excessive accumulation of trigylceride-containing cytoplasmic droplets in peripheral blood smears (referred to as Jordans' anomaly) and other tissues of the body including skin, bone marrow, muscles and cultured fibroblasts. Excessive lipid storage leads to variable forms of clinical presentations including ichthyosis, skeletal and cardiac myopathy and hepatic steatosis. Additionally, some cases have been reported with ataxia, hearing loss, or mental retardation. A recently proposed classification subdivides NLSD into two distinct groups [40]. Depending on whether or not the patients suffer from skin involvement, they are diagnosed with either neutral lipid storage disease with ichthyosis (NLSDI, also known as Chanarin Dorfman Syndrome [MIM #275630] or neutral lipid storage disease with myopathy (NLSDM, [MIM #610717]), respectively. Importantly, this classification finds its molecular basis in the affected genes. Mutations in *ATGL* cause NLSDM, and mutations in *ABHD5* cause NLSDI [41].

On the other hand, polymorphisms and haplotypes in *ATGL* were associated with reduced plasma levels of free fatty acids (FFA) and triglycerides, increased plasma levels of glucose and an elevated risk for type 2 diabetes in the Utah Obesity Case-Control Study [42]. Common haplotypes have also been associated with increased triglycerides in Greenland Inuits [43]. However, no association of rare variants with obesity was observed in a recent case-control study [14].

The aim of this study was to thoroughly investigate the influence of rare variants in the *ATGL* gene on FFA levels in a large healthy working population both by association and biochemical studies. This was achieved by (1) screening the genomic region of the *ATGL* gene for novel variations, (2) in-vitro evaluation of residual enzymatic activity and intracellular localization of all detected protein variants and (3) genetic association studies on FFA levels. All investigations were done in the SAPHIR population, which is an observational study in healthy unrelated Caucasian subjects. Study participants were recruited in the years 1999–2002 by health-screening programs in large companies in and around the Austrian city of Salzburg. For further details see the Methods section below. Our study in a healthy population revealed a pronounced allelic heterogeneity due to mostly rare and even private mutations with, however, modest effects of FFA plasma concentrations.

Besides this screening approach and in order to oppose the mutations found in heterozygote state in a healthy population to an example of a homozygous state, we describe here also the first patient with NLSDM caused by a point mutation within the catalytic dyad (*p.D166G*).

## Results

### Mutation screening

**Figure 2:**
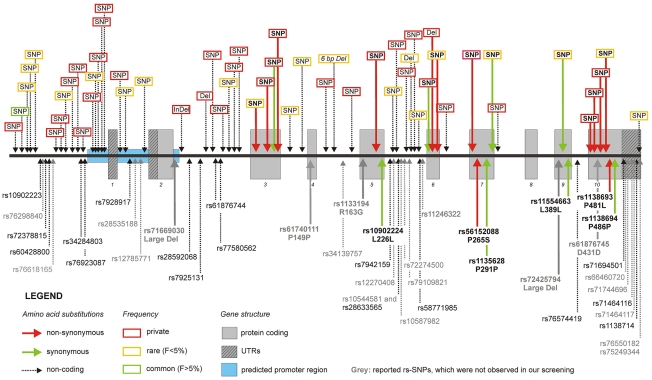
Frequency, type, and localization of all novel variants. The upper part of the figure shows the novel variants, while the lower part depicts previously known SNPs according to dbSNP build 131. Details about the meaning of the color codes and arrow types are given in the legend within the figure. Nearly all genetic variants were rare (yellow boxes) or even private (red boxes).

We screened a total of 11.6 megabases for mutations corresponding to the complete *ATGL* gene region in 1,473 individuals randomly selected from the SAPHIR Study. We detected 20 rare and 34 private variants, but also one common polymorphism, which were not yet reported in dbSNP build 131 (Table 1 and Figure 2). An exhaustive list is given in Table S1. Taken together, rare variants in *ATGL* were present in 7.7% of the screened individuals (n = 113).

**Table 1:** Number of known and newly detected variations.

	Previously known		Our study(only novel variations)
	dbSNP b.131	HapMap Rel.27	
Sum	44	3	55
***Sorted according to localization***			
5′ region/promoter	7	1	16
Introns	18	1	23
3′UTR	9	1	1
Protein-coding region	10	0	15
*non-synonymous*	*4*	*0*	*11*
*synonymous*	*6*	*0*	*4*
***Sorted according to type***			
Single base substitutions	31	3	50
Small deletions and insertions (1 or 2 bp)	3	0	2
Deletions >3 bp and more complex variations	10	0	3

Only about half of the SNPs stored in dbSNP for *ATGL* were detected in our population. However, confirmation of 14 out of 17 independently validated SNPs corroborates the sensitivity of our approach. The inspection of the database entries of the three undetected validated SNPs revealed that two of them were originally detected in samples of different ethnicity than SAPHIR (rs12270408 in Blacks and rs28535188 in Asians) and the third one (rs10544581) was located in a low complexity region.

Although representing a general healthy working population, 39 (2.6%) of the individuals presented rare missense and even nonsense mutations in *ATGL* (Table 2). Most notably, we even identified a heterozygous frameshift variation at aspartate 244, which introduces a premature translation stop at amino acid 254 and thus produces a dysfunctional protein totally lacking lipid droplet binding and catalytic activity (see below). Moreover, two frequent non-synonymous SNPs were present in the population.

**Table 2:** Non-synonymous variants found in 1,473 individuals of the SAPHIR study.

#	Variation		n (Aa/aa)	MAF [%]	dbSNP Accession
*Rare variants*					
1	p.R79Q	c.236G>A	3/0	0.10	ss262967480
2	p.R113H	c.338G>A	1/0	0.03	ss262967482
3	p.H131R	c.392A>G	1/0	0.03	ss262967486
4	p.L219F	c.655C>T	1/0	0.03	ss262967495
5	p.D244fs a	c.732delT	1/0	0.03	ss262967539
6	p.N252K	c.756C>G	26/0	0.88	ss262967509
7	p.P260A	c.778C>G	1/0	0.03	ss262967513
8	p.V402I	c.1204G>A	1/0	0.03	ss262967521
9	p.N426S	c.1277A>G	1/0	0.03	ss262967523
10	p.E437K	c.1309G>A	1/0	0.03	ss262967525
11	p.P477R	c.1430C>G	2/0	0.07	ss262967527
*Polymorphisms*					
12	p.P265S	c.793C>T	79/0	2.28 b	rs56152088
13	p.P481L^c^	c.1442C>T	702/136	28.78 b	rs1138693

**a** Results in a premature stop at amino acid 254.

**b** SNP was genotyped in the complete SAPHIR population (n = 1726).

**c** This SNP is defined in dbSNP as leucine to proline substitution, however, the proline allele is significantly more common. Therefore the SNP is given here as proline to leucine substitution.

The mutation density was highest in the upstream region of *ATGL* (1 every 71 bases) and similar in introns and coding regions (1 every 132 and 115 bases, respectively) (Figure 2 and Table 1). Strikingly, about three quarters of all detected variants in *ATGL* in the present study were until recently unknown.

### Functional studies of *ATGL* variants

**Figure 3:**
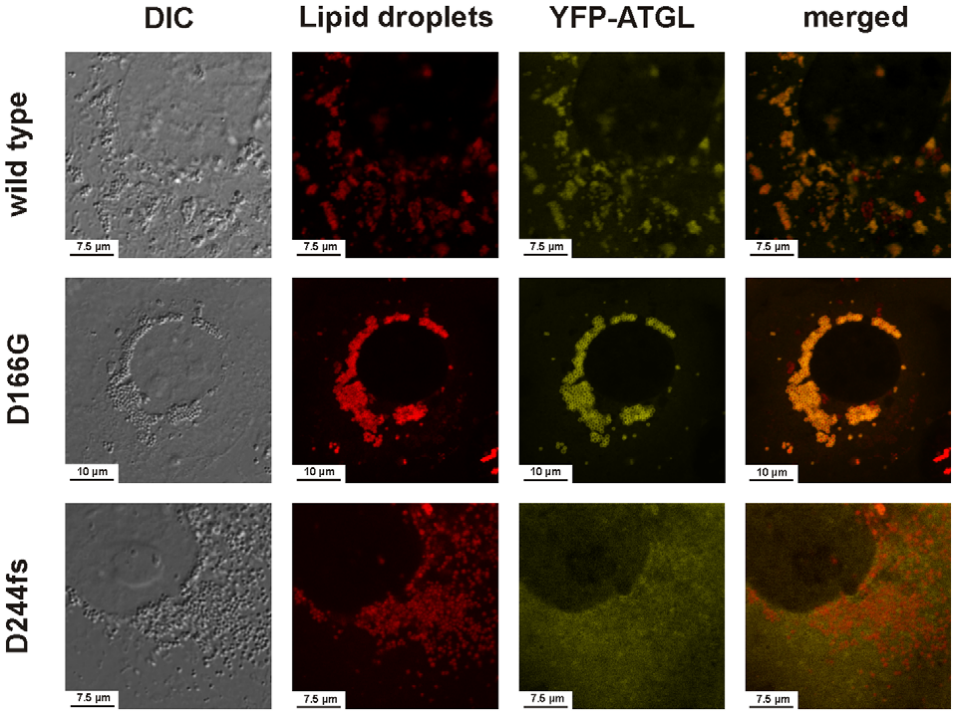
Intracellular localization of the wild-type ATGL protein and the mutants D166G and D244fs as determined by laser scanning microscopy. This image shows the intracellular localization of the YFP-tagged ATGL mutants D166G and D244fs compared to the wild type ATGL in Cos-7 cells loaded with 400 µM fat free BSA-complexed oleic acid to induce lipid droplets generation. Lipid droplets were stained using BODIPY 558/568 C12. While the binding to the lipid droplets was still intact and well pronounced for the D166G mutation, the deletion of the C-terminus in the frameshift mutant D244fs effectively abolished the binding of the protein to the lipid droplets. The merged image clearly shows a diffuse YFP signal in the cytoplasm, which no longer co-localizes with the signal of the BODIPY-stained lipid droplets. The images for all assayed mutants are provided in Figures S1 and S2.

We investigated the intracellular localization and in-vitro triglyceride hydrolase activity of all detected protein variants including the two frequent SNPs rs56152088 and rs1138693 (*p.P265S* and *p.P481L*, respectively). All mutants were readily expressed by Cos-7 cells and, except for the frame shift mutation *p.D244fs,* still correctly localized to the lipid droplets (Figures S1 and S2). In line with previous reports [34], the loss of the C-terminus effectively abolished the binding of the truncated *p.D244fs* protein to the lipid droplets (Figure 3).

**Figure 4:**
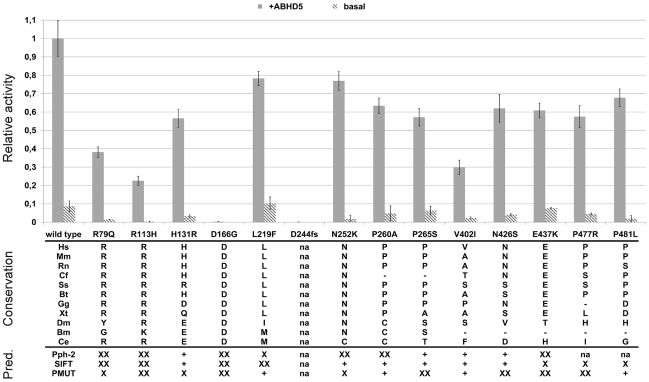
Relative triglyceride hydrolase activities, conservation, and bioinformatic predictions. Triglyceride hydrolase activity: Enzymatic activity is given as relative to wild type *ATGL* after stimulation with murine GST-*ABHD5* and corrected for Cos-7 background activity. Stimulation with *ABHD5* was repeated twice and all measurements were done in triplicates. The activation pattern was consistent for both experiments. Data is presented as mean±standard deviation of the two independent experiments. Conservation panel: *Hs*: Homo sapiens; *Mm*: Mus musculus, *Rn*: Rattus norvegicus, *Cf*: Canis familiaris, *Ss*: Sus scrofa, *Bt*: Bos Taurus, *Gg*: Gallus gallus, *Xt*: Xenopus tropicalis, *Dm*: Drosophila melanogaster, *Bm*: Bombyx mori, *Ce*: Caenorhabditis elegans. na: not applicable (frameshift mutation). Bioinformatic predictions: “XX”: deleterious; “X”: deleterious (low confidence prediction); “+”: benign; “na”: no prediction available; Pph-2: Polyphen-2.

Both basal and *ABHD5*-stimulated catalytic activities showed large variability ranging from total inactivity (*p.D244fs*) over strong impairment (*p.R113H*, *p.V402I*) to nearly wild type activity (Figure 4). All mutants with residual activity were still stimulated by *ABHD5*.

### Identification of the first NLSDM patient with a point mutation in the catalytic dyad

During our screening in the SAPHIR Study, a 20 year old male was referred to our laboratory with the suspicion of NLSDM. Clinical investigations had revealed a dilation of the right cardiac ventricle with severely impaired function, diffuse cardiac fibrosis and vacuolated neutrophiles. However, no ichthyosis was observed. Cultured fibroblasts showed a 1.54-fold accumulation of [9,10-^3^H]-oleate, when compared to 5 healthy controls (51±6.6×10^−3^ vs. 33±5.5×10^−3^ counts per mg protein/24 hours; measurements done in quadruplicates).

In order to corroborate the NLSDM diagnosis, we included the exons and splice sites of *ATGL* of the patient and both parents in our mutation screening, revealing a homozygous *c.497A>G* mutation (*p.D166G*) disrupting the catalytic dyad (Figure 1). Parents were consanguineous and heterozygous for the mutation. Biochemical investigations showed intact intracellular localization, but total loss of catalytic activity (Figure 3 and Figure 4). This is the first proof of the crucial importance of the aspartate 166 in humans, which was so far only demonstrated by in vitro experiments in mice [35], [41].

### Conservation and bioinformatic prediction

Except for valine 402, all mutated amino acids were well conserved in mammals, vertebrates and, partly, even in other bilateria (Figure 4). The catalytic aspartate at position 166 was conserved in all species from man to fruit fly and both asparagine 252 and arginine 113 were conserved in all but one species. However, also several C-terminal variations affected positions were rather conserved in mammals and vertebrates.

The performance of the bioinformatic prediction tools Polyphen-2, SIFT and PMUT was quite ambiguous (Figure 4, bottom). They concordantly predicted a deleterious effect of the N-terminal mutations *p.R79Q*, *p.R113H* and *p.D166G*, but all failed to predict the strong activity decrease of *p.V402I*. The predictions for the C-terminal variations were generally partially inconclusive, probably due to the lower degree of conservation of the C-terminus, its less well defined function and the rather small impact of the variants on the protein function.

### Genetic association studies

**Figure 5:**
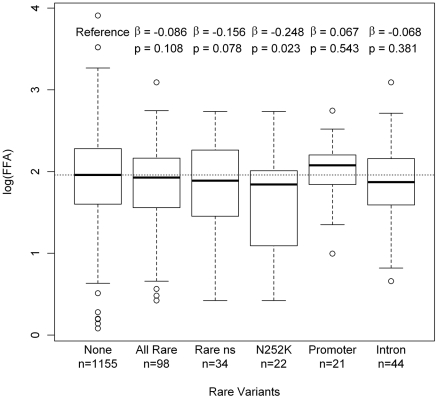
Boxplots on log(FFA) for different classes of rare variants. The figure shows the free fatty acid (FFA) distribution of the carriers of different subgroups of rare variants (all rare variant carriers, carriers of rare non-synonymous mutations, carriers of p.N252K, carriers of variants of rare promoter SNPs (defined as SNPs upstream of *ATGL* or within the predicted promoters structures in intron 1, see also Figure 2) and carriers of rare intronic SNPs) compared to individuals without rare variants (labeled with „none“). Variants in *ATGL* showed a tendency towards lower FFA levels, which was most pronounced for the p.N252K group. Conversely, a tendency towards higher FFA levels was observed for variants in the putative promoter region. Estimates and p-values were calculated by linear regression on the logarithmized FFA levels (expressed as mg/dl).

Finally, we aimed to evaluate the genetic association of rare variants with FFA levels in the SAPHIR population using both a continuous and an “extreme phenotype” approach. FFA levels were available for a subgroup of 1253 individuals out of the 1473 individuals screened for variants. Figure S3 shows the distribution and corresponding percentiles of the FFA levels. Effect estimates from a linear model on log(FFA) for each of the rare variants showed an accumulation of increased plasma levels for variants in the promoter region (Figure S4). For the other classes of rare variants the effect direction was rather inconclusive, suggesting a rather heterogeneous effect of rare *ATGL* variants on the population if present in heterozygous state. Overall, FFA levels showed a slight shift towards lower FFA values for rare variant carriers, which seemed to be primarily driven by the missense variant p.N252K (p = 0.023) (Figure 5). No clear association of any other missense variant was observed.

**Figure 6:**
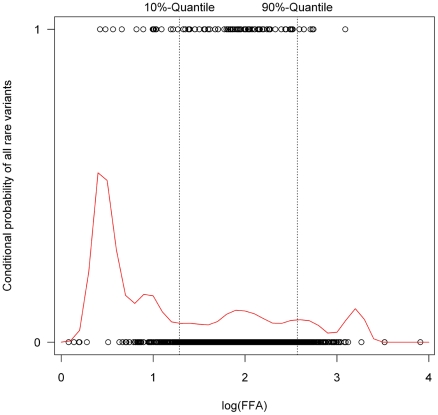
Conditional density plot showing the probability of carrying a rare variant conditional on free fatty acid (FFA) plasma concentrations. The dots at the upper, respectively lower border of the plot depict the log(FFA) value (given on the X-axis) of each proband with (upper dots) or without (lower dots) rare variants. Based on the observed distribution, the curve gives the probability of carrying a rare variant conditional on FFA values. This was clearly higher in the lower 10% quantile, indicating an accumulation of rare variants in the lower 10% quantile. The position of the upper and lower 10% quantiles of the log(FFA) in the screened samples are marked by the dotted lines.

Next we assessed the impact of rare *ATGL* variants in the phenotypic extremes, using the lower and upper 10%-quantiles of FFA values. The conditional density plot (Figure 6) showed an excess of rare variants in the lower tail of the FFA distribution. It indicates that using the bottom and top 10% of the distribution provides a good discrimination between individuals with at least one or no rare variants without losing too many data and therefore information.

**Figure 7:**
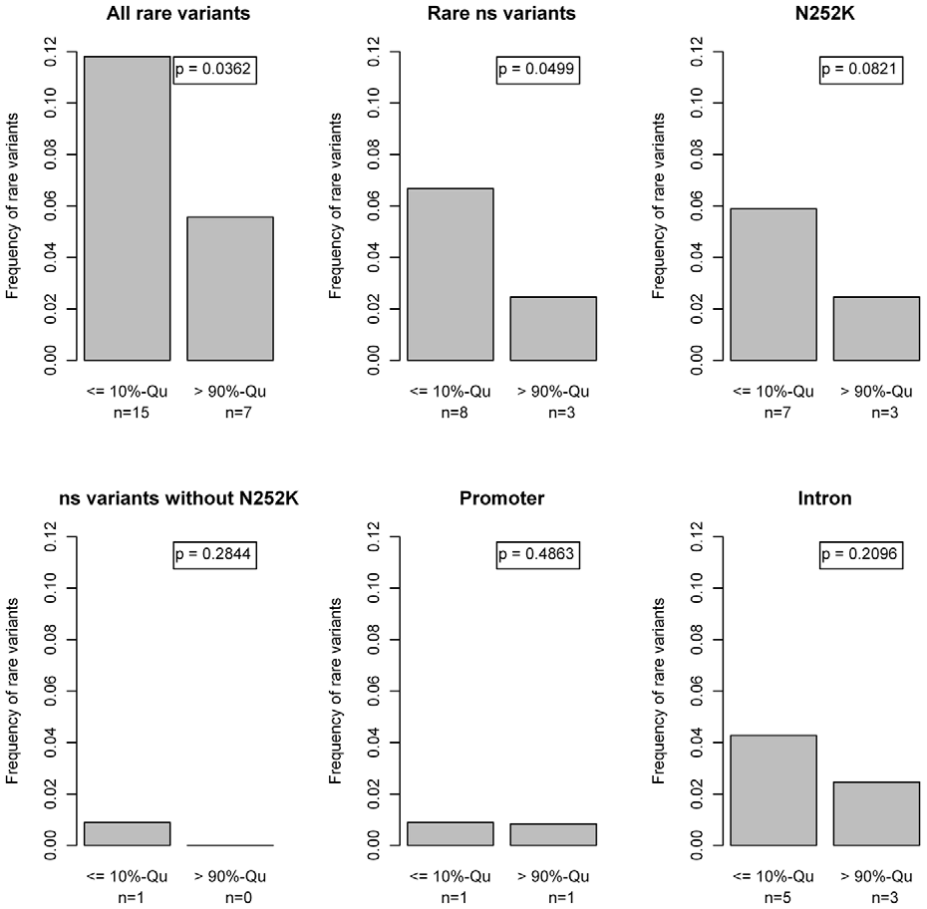
Barplots and beta tests comparing the first and last 10%-quantiles of the free fatty acid (FFA) distribution. Differences in the proportion of rare variant carriers between the lower and the upper 10% quantiles of the FFA levels. We observed an accumulation of rare variants in the lower 10% quantile, which was mainly driven by the p.N252K variant with a contribution of rare intronic variants. No accumulation of other missense variants or promoter variants was observed. Statistical significance was assessed with the Beta-Test proposed by Li et al [44]. “ns”: non-synonymous.

Fifteen rare variants (11.8%) were found in the group of low FFA values compared to 7 (5.6%) in the group of high FFA values. This difference in proportions was statistically significant (p = 0.036; Beta-test [44]). Especially *p.N252K* and, surprisingly, variants in the introns were contributing to this excess of rare variants in the lower FFA quantile (Figure 7).

Table 3 gives a comparison of potential confounders between the two tail groups. However, none of these parameters were associated with both FFA levels and rare variants (data not shown), therefore excluding confounder effects.

**Table 3:** Descriptive characteristics of individuals analyzed in the association studies.

	Alln = 1253	FFA ≤10% Quantile (3.59 mg/dl), n = 127	FFA >10% Quantile (12.83 mg/dl), n = 126
Sex (male/female), n (%)	827/421 (66/34%)	120/7 (94.5/5.5%)	47/79 (37.3/62.7%)
Age (years)	51.2±5.9	48.4±5.7	53.5±5.2
Body Mass Index (kg/m^2^)	26.9±4.1	25.6±3.0	27.8±5.4
Waist circumference (cm)	94.7±12.3	92.9±8.8	94.7±15.6
Diabetes mellitus, n (%)	42 (3.4%)	2 (1.6%)	6 (4.8%)
Fasting insulin (mmol/L)	7.5±5.3	6.3±4.1	8.9±6.1
Glucose (mg/dl)	93.9±18.7	89.7±9.4	97.6±20.4
HOMA Index	1.83±1.60	1.48±1.14	2.14±1.64

Data are provided for the entire group as well as for the two tail groups of the free fatty acid (FFA) distribution. FFA levels were available for a total of 1,253 individuals of the 1,473 subjected to mutation screening.

## Discussion

### Mutation screening in SAPHIR and functional evaluation of missense variants

We investigated the influence of rare genetic variants in *ATGL* on FFA levels by an unbiased approach in a healthy working population by genetic association studies followed by functional investigations. The biochemical characterization of the detected missense variants demonstrated the presence of several mutated but still operative alleles with largely varying residual activity even in a healthy population. However, one variant showed a total loss of function (*p.D244fs*) and three variants presented markedly impaired catalytic activities (p.*R79Q*, *p.R113H* and *p.V402I*).

The effects of p.D244fs and p.V402I were against our expectations. The *p.D244fs* variant represents the shortest human nonsense mutation, which has been functionally investigated so far. Unlike most previously described forms of truncated ATGL [34], [35], *p.D244fs* totally lacked catalytic activity, probably due to critical changes in the overall protein structure produced by a frameshift in the α/β hydrolase fold. A similar finding for a synthetic 178X construct was recently described by Kobayashi et al. [45].

The distinct activity decrease of *p.V402I* was puzzling, since V402 is a C-terminal, non-conserved amino acid. However, most neighboring amino acids appear to be highly conserved (Figure S5), thus suggesting a still unknown regulatory role of this amino acid stretch. Intriguingly, Duncan et al. recently reported the binding of still unidentified proteins to the C-terminus of murine ATGL [35].

### Genetic association studies

Our population-wide association studies on FFA levels in the SAPHIR Study were rather inconclusive and showed mostly non-significant trends towards lower FFA levels for rare variant carriers. These trends were primarily driven by the *p.N252K* variant (Figure 5). Heterozygous carriers of this variant showed a decrease in mean FFA levels of 15% compared to the wild type (6.70 mg/dl vs. 7.86 mg/dl). This is in line with previous results in the Utah Obesity Case Control study, where we observed a decrease of about 12% [42]. Interestingly, *p.N252K* did not show a pronounced decrease in enzyme activity, although it affects one of the best conserved amino acids among all evaluated variants (Figure 4). This suggests that the association of *p.N252K* with FFA levels may be based on mechanisms other than the mere modification of the catalytic activity. On the other hand, given the nearly wild type-like residual activity and the lower conservation grade of the affected amino acids, the lack of association of the more frequent variations *p.P265S* and *p.P481L* seems well plausible.

Since no clear effect of rare variants on the whole population was observed we next investigated the impact of rare variants in the outer quantiles of the FFA distribution. This uncovered an excess of rare variants in the lower 10%-quantile, which was to a large extend driven by *p.N252K*. However, the accumulation of rare variants was even more pronounced when considering all rare variant carriers, due to a contribution of rare intronic variants (Figure 7). On the other hand, no accumulation of other missense variants was seen.

Since enzyme variants were frequently shown to follow a recessive model, the observed lack of significant associations of *ATGL* variants with FFA levels may indicate possible recessive effects of missense mutations in *ATGL*. This was also observed in NLSDM carriers [40]. Accordingly, even the heterozygote carrier of *p.D244fs* did not present any abnormalities in FFA levels (Figure S4).

### Patient with NLSDM due to a point mutation in the catalytic dyad

NLSDM-causing mutations were frequently reported to be located in the C-terminus, thus keeping intact the patatin domain and the catalytic dyad [33]. Only two mutations in the patatin domain have been described so far: a retrotransposon insertion into exon 3 [46] and a frameshift mutation in exon 4 (*p.Q160fs*) [47], [48]. The latter also causes a mutation of the active site D166, but possible effects on enzyme inactivation were not investigated [33]. We describe the first patient with a point mutation of the catalytic aspartate (*p.D166G*), which is sufficient to effectively abolish the catalytic activity (Figure 4), despite of a still intact binding to the lipid droplets (Figure 3). The seemingly well pronounced binding of the D166G reminds to the recently reported *p.P195L* loss of function mutation [40], which showed an slightly increased binding to lipid droplets too [34], which might be caused by a decreased protein turn-over rate. In line with previous reports [40], [45], [48], the patient aged 19 presented a severe cardiomyopathy and is currently awaiting a cardiac transplant. This confirms the potentially highly deleterious effects of functionally impaired *ATGL* alleles.

Although both the muscles and the heart are not classical lipid storage tissues, the observation of myopathy and cardiomyopathy is in line with observations in *ATGL-ko* mice, which die from cardiac insufficiency at about week 12 from birth and show reduced exercise tolerance [33], [49]. Recent studies have provided some insights in the underlying mechanisms [33], [49], [50]. By using mice lacking *ATGL* in all tissues except the cardiac muscle, Schoiswohl et al have demonstrated a crucial role of *ATGL* for the supply of the working muscle with fatty acids [50]. Conversely, the cardiomyopathy is thought to be produced by a defect in the utilization of FFAs by the cardiomyocytes [33]. The cardiac muscle is not able to autonomously synthesize fatty acids, but rather relies on the exogenous supply from plasma. The uptaken FFAs are however not directly utilized for β-oxidation, but first re-esterified to triglycerides, which, in case of an *ATGL* deficiency, then accumulate in the cardiomyocytes. This issue is further exacerbated by the fact that the FFA uptake machinery still remains fully induced, despite the impaired triglyceride breakdown [33]. This may then produce the observed cardiac hypertrophy and subsequent cardiac failure.

### Strengths and limitations of our study

The resequencing of individuals with extreme phenotypes has been very successful to identify new functional variants and shed light on the molecular function of involved proteins [20]–[22], [51]. However the total impact of rare variants on traits in healthy populations is still unclear. The rare variant hypothesis has been repeatedly invoked to explain at least partially the missing heritability issue [1], [7]–[9], and postulates a high allelic heterogeneity consisting of individually rare but collectively common rare variants [3]. However, the well established impact of rare variants on the tails of the phenotypic distribution alone may not sufficiently explain a considerable portion of heritability and current expectation might be too optimistic [23]. Therefore, besides the highly deleterious rare variants in the outmost tails, also a number of rare variants with modest but still detectable effects should exist. These effects may, however, not be strong enough to drive their carriers into the outmost tails of the distribution, but rather contribute to the overall genetic distribution.

The main strengths of our study are (1) the unbiased approach based on a large healthy working population, (2) the functional follow-up of all detected missense variants, and (3) the statistical evaluation using both on a continuous and dichotomous approach. Only a few similar studies have been carried out before [13], [15], [20], and none has so far evaluated also intronic and promoter variants.

However, since a healthy population rather reflects the general variability in the population and will likely not be enriched for functional variants, the size of our screening population could be considered a limitation of our study, as it bears the risk of underestimating the role of rare variants due to insufficient statistical power. Due to lack of power, statistical tests were not corrected for multiple testing. Thus, the association results presented can merely be seen as a trend, which has to be followed in further, even larger studies.

Finally, since coding mutations are most prone to exert a severe impact on the protein function, we restricted the functional evaluation only to coding variants, providing the so far largest catalog of functionally investigated *ATGL* variants. However, the additional exhaustive investigation of all non-coding variants detected was beyond the scope of this study. Nevertheless, we are well aware that also non-coding variants can exhibit strong influence the phenotype [52]. Therefore, we cannot definitely rule out the presence of unidentified regulatory variants.

### Conclusions

Although *ATGL* represents a crucial player in the lipolysis cascade, the role of rare genetic variants in *ATGL* remains elusive. We found several rare genetic variants and observed a trend towards lower FFA levels in carriers, as wells as an accumulation of rare variants in the lower 10%-quantile of FFA levels. However, the effects were rather small and suggested a secondary role of rare *ATGL* variants on the FFA levels in a healthy population.

Our study demonstrates that even in a healthy population, rare variants are collectively common and provide a large amount of allelic heterogeneity. However, at least in our study, their role on a quantitative trait in a healthy population outside of phenotypic extremes was rather small. Whether this implies that the impact of rare variants is, more generally, limited to the outer percentiles of the phenotypic distribution of a quantitative trait, remains to be investigated in further studies.

## Methods

### SAPHIR population

SAPHIR (Salzburg Atherosclerosis Prevention program in subjects at High Individual Risk) is an observational study involving 643 females aged 50 to 70 years and 1083 males aged 40–60 recruited between 1999 and 2002 in large companies in and around the Austrian city of Salzburg [53]. The differential age range between the sexes was chosen to match the cardiovascular risk, which is lower for women but matches the risk of men aged 10 years plus. All subjects were unrelated and of Caucasian origin. Exclusion criteria were severe obesity (BMI >40 kg/m^2^), established coronary artery, cerebrovascular or peripheral arterial disease, congestive heart failure, valvular heart disease, chronic alcohol (more than three drinks/day) or drug abuse and pregnancy. At baseline, a detailed personal and family history of all study participants was assessed via standardized questionnaires and anthropometric parameters were measured in the course of a physical examination. Venous blood was collected after an overnight fast and plasma samples were either used immediately for analysis or were stored frozen at −80°C. Plasma FFA concentrations were measured by using a colorimetric assay (Wako, USA) and DNA was extracted from total blood using the Invisorb Blood Universal Kit (Invitek, Germany). Informed consent was obtained from each participant and the study was carried out in accordance with the local ethics committee.

### Patient with NLSDM due to a point mutation in the catalytic dyad

A 19-year-old male of consanguineous South Asian parents, who was previously well and has no significant family history apart from an older sister who died of an unknown cardiac problem at the age of 9 (no post-mortem was carried out) presented to his local Accident and Emergency Department with acute episode of shortness of breath and generally unwell. He was subsequently admitted under the care of cardiology team for further investigations. He had normal developmental milestones, was not on any medications and was due to enroll first year third level education. On admission, he had normal heart rate and blood pressure, his body mass index was below 25 kg/m2. Routine blood tests including full blood count, renal, bone profiles and creatinine kinase (CK) were all normal. His liver function tests were also essentially normal apart from mildly raised γ-glutamyl transferase (GGT) suggestive of mild fatty liver which was confirmed on a liver ultrasound. No ischaemic changes were noted on 12-lead electrocardiogram (ECG). An echocardiogram showed severely dilated both ventricles with left ventricle end-diastolic diameter of 72 mm (normal <49±4 mm) and ejection fraction of 14% (normal >58±7%). He denied any musculo-skeletal symptoms and no history of ichthyosis was reported in the patient or the family.

He subsequently developed weakness of his left arm associated with impaired co-ordination, and brain magnetic resonance imaging showed a small cardio-embolic infarct in the right parietal and cerebellum areas. 24 hr electrocardiogram showed episodes of non-sustained broad complex tachycardia and multiple ventricular ectopics. He was subsequently warfarinised and has had implantable cardioverter defibrillator (ICD) and cardiac resynchronization therapy (CRT) inserted, and being placed on cardiac transplant waiting-list. General metabolic and autoimmune screenings for cardiomyopathy were negative apart from Jordan's anomaly observed on peripheral blood smear histology, suggestive of a neutral lipid storage disorder.

### Detection of genetic variants

Mutation screening was performed using the Ecotilling technology. Ecotilling is a mutation detection technology based on mismatch cleavage and enables sensitive high throughput detection of rare mutations in 1.5 kb large PCR fragments and up to 768 pooled samples per run [54]. It therefore dramatically lowers the costs for rare variant screening in large populations by introducing a cost-effective and robust prescreening step capable of both detecting and exactly localizing several mutations per amplicon and therefore significantly easing the subsequent follow-up steps. Moreover, the intrinsic double detection of each mutation ensures high specificity [54]. A flowchart detailing the Ecotilling process is provided in Figure S6. Briefly, Ecotilling screening was performed as described before with minor modifications [54] and using fourfold pooled DNA. The genomic region of *ATGL* (1506 bp before exon 1 to 106 bp after end of exon 10) was amplified in 8 overlapping PCRs using LI-COR IRDye labeled primers (Metabion GmbH, Germany). After heteroduplex formation (99°C for 10 min, 70 cycles: 70°C for 20 s, decreasing 0.3°C per cycle), 4 µl PCR product were incubated at 45°C with 1.5 µl reaction buffer (1 M MgSO_4_, 0.1 M HEPES pH 7.0, 0.1 M KCl, 0.02% Triton X-100, 2 mg/ml BSA), 0.1 µl purified *Cel-I* (diluted 1∶10,000) and 9.4 µl PCR grade water (Lonza, Belgium). The digestion was stopped with 5 µl 75 mM EDTA, purified by Sephadex G50 (GE Healthcare, Sweden), mixed with 5 µl of formamide loading buffer and reduced to approximately 1.5 µl at 85°C. 0.8 µl were then loaded on the LI-COR 4300 DNA Analyzer (LI-COR Biosciences, USA). The assay design and evaluation approach was described in detail earlier [55]. For primer sequences and PCR conditions see Tables S2, S3, S4, S5. The Ecotilling approach was applied in 1473 randomly selected individuals from the SAPHIR Study.

Each rare mutation was then confirmed in each affected sample by sequencing. Common SNPs present in multiple samples were confirmed in at least one sample to ensure the identity of the respective Ecotilling signal. Sequencing was performed using on an Applied Biosystems (ABI) 3130xl Genetic Analyzer the BigDye Terminator v1.1 cycle sequencing kit (ABI Applied Biosystems, USA) using a modified protocol. For details see Tables S4 and S5. Genotyping of rs56152088 (p.P265S) and rs1138693 (p.P481L) in the entire population was performed with an ABI 7900HT Fast Real-Time PCR System using ABI Taqman assays.

### Site-directed mutagenesis

Point mutations were inserted into the eukaryotic expression vector pEYFP-C1 (BD Biosciences Clontech, USA) encoding the sequence of human *ATGL* and a yellow-green fluorescent protein (YFP) [34] using the Invitrogen GeneTailor Site-Directed Mutagenesis System. Fidelity of picked clones was verified by restriction enzyme digestion and bidirectional sequencing. PCR conditions and primer sequences are given in Tables S6, S7, S8.

### Expression of human ATGL mutant proteins

Expression of recombinant YFP-ATGL proteins and controls (wild type ATGL and β-galactosidase (*LacZ*)) was performed in Cos-7 cells (ATCC CRL-1651; ATCC, USA). Cos-7 cells were cultured at 37°C, 7.5% CO_2_ and 95% humidity in standard growth medium consisting of Dulbecco's Eagle modified medium (GIBCO, USA) supplemented with 10% fetal calf serum, (Sigma, USA), 100 µg/ml Streptomycin and 100 U/ml Penicillin (GIBCO). For transfection, cells were seeded at a density of 9*10^5^ cells/10 cm^2^ dish, cultured in standard growth medium for 24 hours and transfected in serum-free medium with 6 µg plasmid DNA using Metafectene (Biontex, Germany).

### Isolation of cytoplasmatic extracts for triglyceride hydrolase assay

After 48 hours in regular growth medium, cells were collected in PBS buffer, pelleted at 290 g for 3 minutes and resuspended in assay buffer (0.25 M sucrose, 1 mM EDTA, 1 mM DTT, 20 µg/ml leupetine, 2 µg/ml antipain, 1 µg/ml pepstatin, pH 7.0). All further steps were performed on ice. To generate cytoplasmatic extracts, cells were disrupted by sonication (Sonicator 4000 with Microtip Probe, Qsonica LLC, USA) and centrifugated at 1000 g (5 min, 4°C). Total protein concentration was determined by a colorimetric assay (Protein Assay, Bio-Rad Laboratories, USA) with BSA as standard.

### Western blot analysis

The expression levels were determined by Western blotting using 20 µg cytoplasmatic extract, a rabbit anti-GFP antibody (1∶5,000) (Abcam, USA) and a goat anti-rabbit IgG antibody conjugated with horseradish peroxidase (1∶10,000) (Vector Laboratories, USA). Chemoluminescence was induced using the GE Amersham ECL Plus Kit and detected with a Typhoon 9400 Imager (GE Healthcare). Relative expression rates were determined with the ImageQuant Software 5.2 (GE Healthcare) using the local median method for background correction (Figure S7). To exclude the saturation of the gel bands, which might affect correct quantification, amounts from 9 to 50 µg protein extract of the lysate with the highest expression levels (*p.V402I*) were blotted without reaching signal saturation (data not shown).

### Triglyceride hydrolase assay

Triglyceride hydrolase activity was determined as the amount of FFA released by 15 µg cytoplasmatic extract from a radiolabeled substrate containing [9,10-^3^H]-triolein in 1 hour [34], [56]. The wild type protein and each mutant were tested for basal and *ABHD5* stimulated activity. Determination of ABHD5 stimulated activity was measured by adding purified murine GST-ABHD5 [36]. The concentration of ABHD5 leading to maximal stimulation of ATGL was determined before. All measurements were done in triplicates and corrected for Cos-7 background activity and varying expression levels (see Figure S7).

### Intracellular localization

Cos-7 cells were seeded on microscope slides in six well plates at a density of 1.5*10^5^ cells/well and transfected with 1 µg DNA per well. 24 hours after transfection, cells were incubated for 20 hours with regular growth medium containing 400 µM fat free BSA-complexed oleic acid to induce lipid droplet generation. Lipid droplets were finally stained by incubating the cells with 1 µg/ml Bodipy 558/568 C12 (Invitrogen, USA) for 1 hour. The localization of the YFP-tagged ATGL mutants was determined by laser scanning microscopy using a Leica SP5 confocal microscope (Leica Microsystems, Germany). YFP fluorescence was excited at 514 nm and emission was detected between 520 and 540 nm. BODIPY 558/568 C12 was excited at 561 nm and detected between 565–600 nm (Figures S1 and S2. Selected mutants are shown in Figure 3).

### Lipid accumulation in p.D166G fibroblasts of the patient with NLSDM

Cultured fibroblasts were incubated as monolayers in multi-well plates in Kreb's ringer pH 7.0, 220 µmol/L [9,10-3H]oleic acid complexed to α-cyclodextrin (25mg/ml) for 5 hours at 37°C. The supernatant was removed, cells were washed in phosphate buffered saline and digested with 1N sodium hydroxide. Total accumulated tritium counts in the incubated cell digests were measured by liquid scintillation counting in patient and simultaneous normal controls and expressed in relation to fibroblast protein.

### Statistical and bioinformatic analysis

For each of the variants, linear regression was performed on the logarithmized FFA values. To strengthen the power of the analysis including rare variants, they were grouped into carriers of rare variants (“all rare variants”) and non-carriers. This group was refined to several subgroups, including carriers of non-synonymous variants, variants in the promoter region, intronic variants and carriers of p.N252K. To follow the hypothesis that rare variants contribute to the “extremes” of a phenotype, FFA values were a priori categorized into their lower and upper 10% quantiles. The choice of this cutpoint was evaluated by a conditional density plot showing the probability of rare variants conditional on FFA values (Figure 6).

Proportion differences of rare variants between these extreme groups of FFA were tested using the Beta-Test proposed by Li et al [44].

All amino acid mutations were submitted to Polyphen-2 using the HumDiv dataset [57], SIFT [58] and PMUT [59]. Evaluation of non-coding SNPs was carried out as described before [60]. Promoter prediction was done with the GenomatixSuite PE (Genomatix GmbH, Germany). Conservation was evaluated using ClustalW [61] on the Uniprot Homepage (http://www.uniprot.org). Accession numbers of the sequences used are given in Table S9.

## Supporting Information

Figure S1 Intracellular localization of wild-type ATGL protein and mutants p.R79Q, p.R113, p.H131R, p.D116G, p.L219F, p.D244fs, and p.N252K. For each mutant differential interference contrast image, Bodipy 558/568 C12 stained lipid droplets, YFP-tagged ATGL proteins and merged fluorescence images are shown. According to previous reports, the deletion of the C-terminus in the frameshift mutant D244fs abolished the binding to the lipid droplets. The merged image clearly shows a diffuse YFP signal in the cytoplasm, which no longer co-localizes with the signal of the Bodipy-stained lipid droplets. All other missense variants were however still able to correctly bind to the lipid droplets.
(3.48 MB TIF)

Figure S2 Intracellular localization of mutants p.P260A, p.P265S, p.V402I, p.N426S, p.E437K, p.P477R, and p.P481L. For each mutant differential interference contrast image, Bodipy 558/568 C12 stained lipid droplets, YFP-tagged ATGL and merged fluorescence images are shown. For p.P265S, p.V402I, p.E437K and p.P481L no DIC images were available. Transmission light images are shown instead.
(2.98 MB TIF)

Figure S3 Histogram showing the distribution and percentiles of the FFA levels in the SAPHIR samples used for association studies. FFA levels are given in mg/dl.
(0.20 MB TIF)

Figure S4 Beta dot plot. This figure shows the distribution of effect estimates from the linear model on log(FFA) for each of the rare variants independently. The diameter of the circles corresponds to the frequency of the variants (see Table S1). An accumulation of positive effects for promoter variants (green points) was observed, while the other classes of variants did not show any common direction in their effects.
(0.33 MB TIF)

Figure S5 Alignment of the C-terminal region surrounding V402 (screenshot of the ClustalW tool on the Uniprot homepage). The position of V402 is marked by the red rectangle. The protein sequence immediately before V402 is highly conserved in mammals and vertebrates.
(1.43 MB TIF)

Figure S6 Description of the Ecotilling process. STEP 1: The gDNA is normalized to 20 ng and arrayed on 96 well plates. STEP 2: Every four plates are pooled and normalized to 10 ng. STEP 3: The pooled DNA is used as a template for PCR amplification of the region of interest, employing primers labeled with IRDye 700 for the forward primer (red star), respectively the IRDye 800 for the reverse primer (blue star). STEP 4: The PCR fragments are heat-denatured and slowly renatured in order to allow the formation of heteroduplexes of double-stranded DNA from different individuals. In case that a SNP is present in this fragment, a subpopulation of double-strands will contain a mismatch, which will be cut by the Cel-I endonuclease in the next step. The amplification products are then partially digested with the endonuclease Cel-I which recongises DNA mismatches with high specificity and cuts on their 3′ end. This produces for each mismatch (respectively SNP) two digestion fragments of complementary length and with different labels on their 3′ end. STEP 5: The digestion products are size-fractionized using in a denaturing PAGE and both wavelengths are detected simultaneously. Each SNP produces two additional bands, besides the signal of the undigested full-size product, whose length sums up to the length of the full-size product. Each SNP is doubly detected and therefore quality controlled.
(0.52 MB TIF)

Figure S7 Western blot analysis of the Cos-7 cell lysates used for triglyceride hydrolase assays. Relative expression levels and correction factors for triglyceride hydrolase activities were determined as described in the main text and are given in the table below. Coomassie stained blots are shown for loading control.
(1.26 MB TIF)

Table S1 New variations detected in the SAPHIR population.
(0.11 MB DOC)

Table S2 PCR conditions for Ecotilling screening (LI-COR IRDye-labeled).
(0.04 MB DOC)

Table S3 PCR conditions for unlabeled amplification.
(0.04 MB DOC)

Table S4 Cycling and sequencing protocols for ATGL Ecotilling.
(0.04 MB DOC)

Table S5 Amplification (A) and sequencing primers (S) for the ATGL gene region.
(0.16 MB DOC)

Table S6 PCR conditions for site directed mutagenesis.
(0.05 MB DOC)

Table S7 Cycling conditions for site directed mutagenesis.
(0.03 MB DOC)

Table S8 Primer sequences for site directed mutagenesis.
(0.04 MB DOC)

Table S9 GenBank Accession numbers of the proteins used for conservation analysis.
(0.04 MB DOC)
