# Supplementary figures and images for: Investigation and Functional Characterization of Rare Genetic Variants in the Adipose Triglyceride Lipase in a Large Healthy Working Population
Source: PLoS Genet. 2010 Dec 9;6(12):e1001239. doi: 10.1371/journal.pgen.1001239 (PMC3000363; doi:10.1371/journal.pgen.1001239)

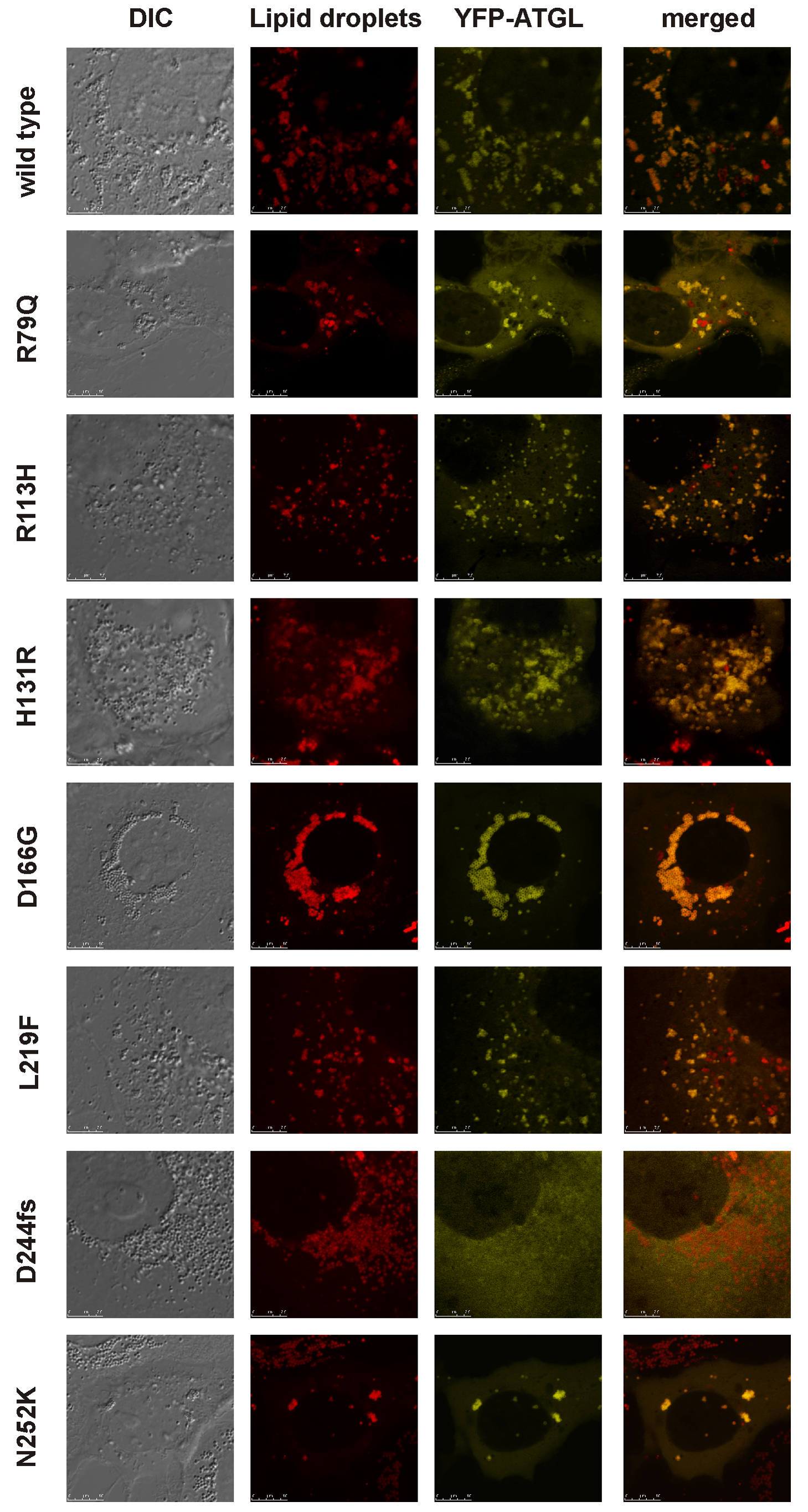

Supplement: Figure S1 — Intracellular localization of wild-type ATGL protein and mutants p.R79Q, p.R113, p.H131R, p.D116G, p.L219F, p.D244fs, and p.N252K. For each mutant differential interference contrast image, Bodipy 558/568 C12 stained lipid droplets, YFP-tagged ATGL proteins and merged fluorescence images are shown. According to previous reports, the deletion of the C-terminus in the frameshift mutant D244fs abolished the binding to the lipid droplets. The merged image clearly shows a diffuse YFP signal in the cytoplasm, which no longer co-localizes with the signal of the Bodipy-stained lipid droplets. All other missense variants were however still able to correctly bind to the lipid droplets. (3.48 MB TIF) [file pgen.1001239.s001.tif]

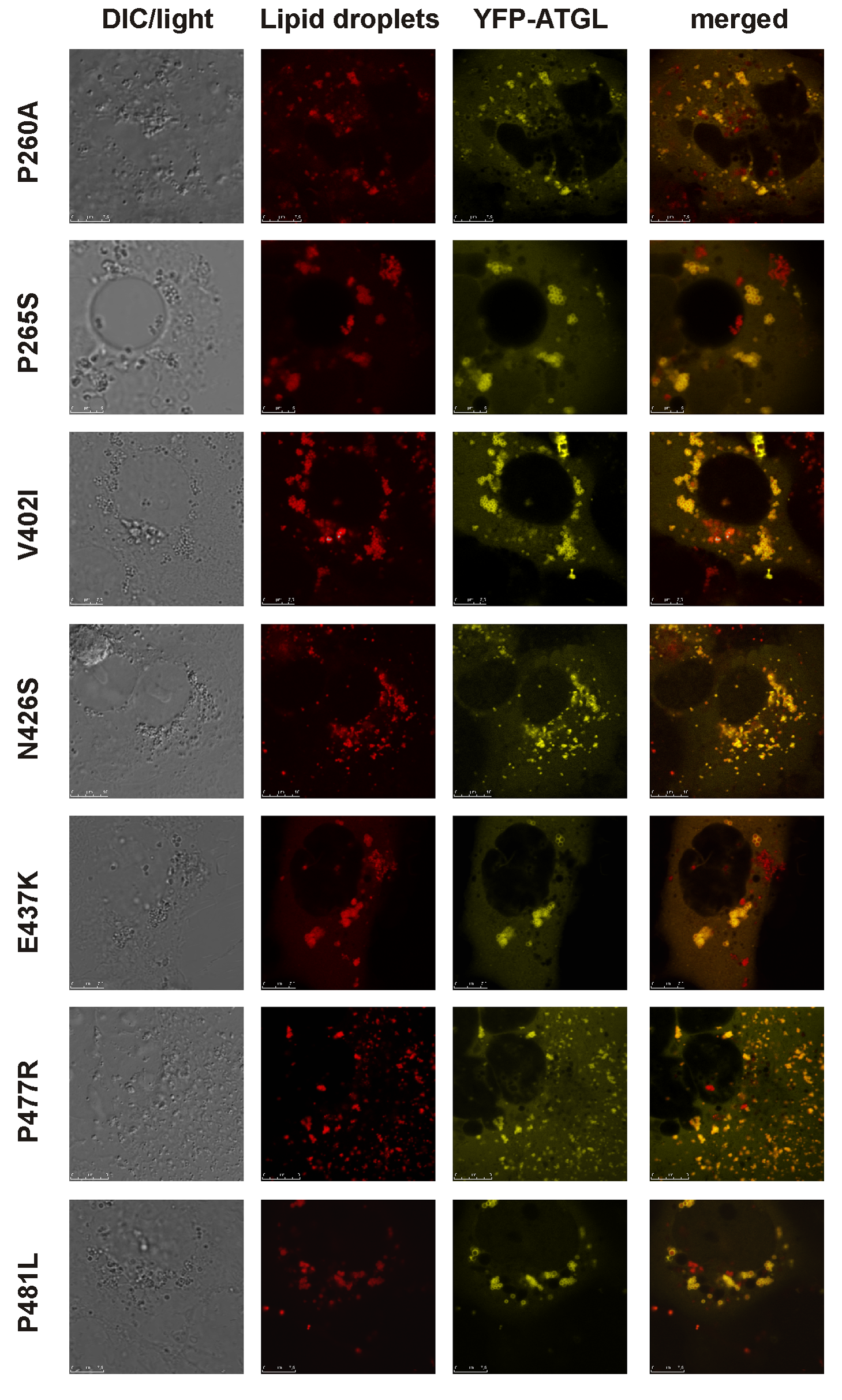

Supplement: Figure S2 — Intracellular localization of mutants p.P260A, p.P265S, p.V402I, p.N426S, p.E437K, p.P477R, and p.P481L. For each mutant differential interference contrast image, Bodipy 558/568 C12 stained lipid droplets, YFP-tagged ATGL and merged fluorescence images are shown. For p.P265S, p.V402I, p.E437K and p.P481L no DIC images were available. Transmission light images are shown instead. (2.98 MB TIF) [file pgen.1001239.s002.tif]

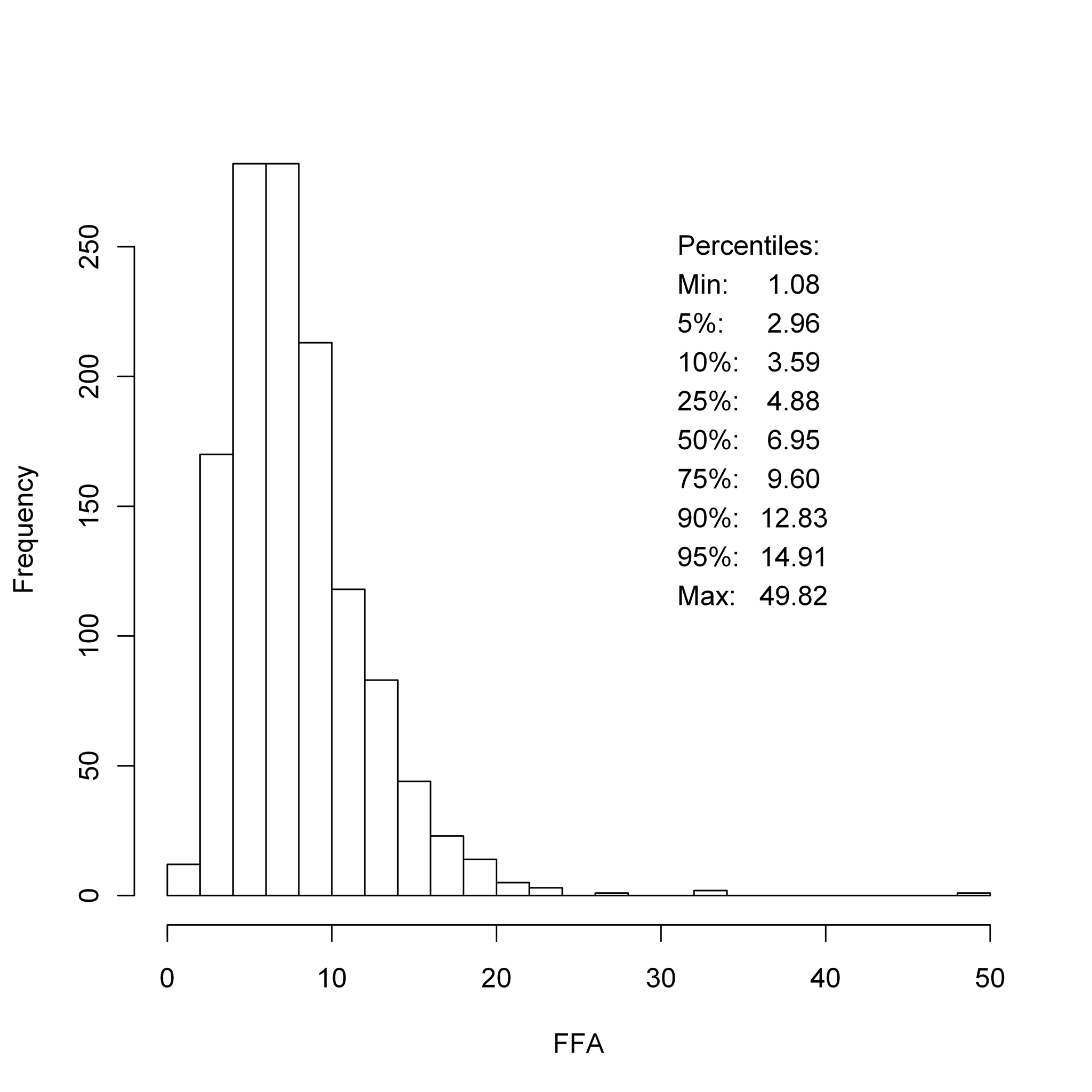

Supplement: Figure S3 — Histogram showing the distribution and percentiles of the FFA levels in the SAPHIR samples used for association studies. FFA levels are given in mg/dl. (0.20 MB TIF) [file pgen.1001239.s003.tif]

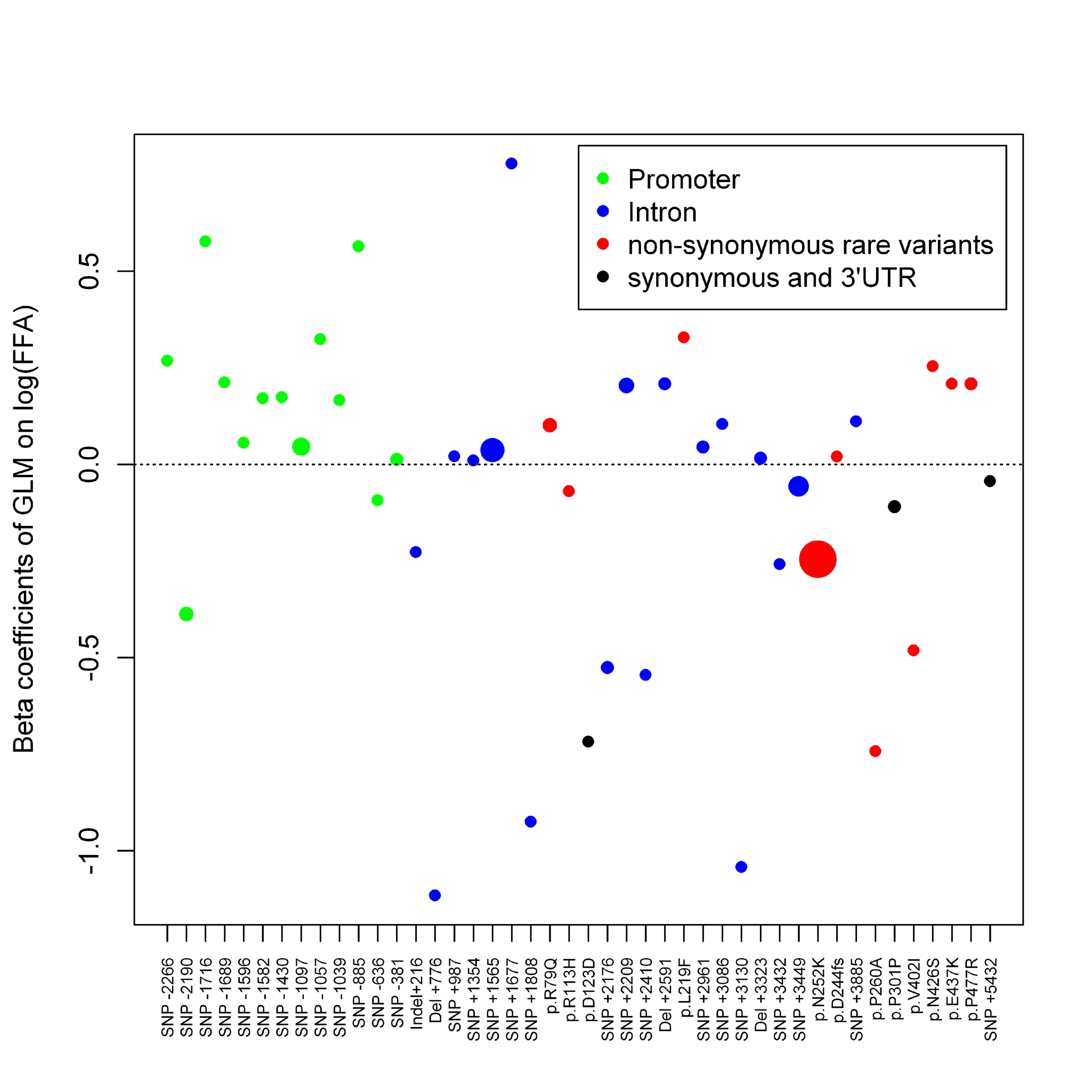

Supplement: Figure S4 — Beta dot plot. This figure shows the distribution of effect estimates from the linear model on log(FFA) for each of the rare variants independently. The diameter of the circles corresponds to the frequency of the variants (see Table S1). An accumulation of positive effects for promoter variants (green points) was observed, while the other classes of variants did not show any common direction in their effects. (0.33 MB TIF) [file pgen.1001239.s004.tif]

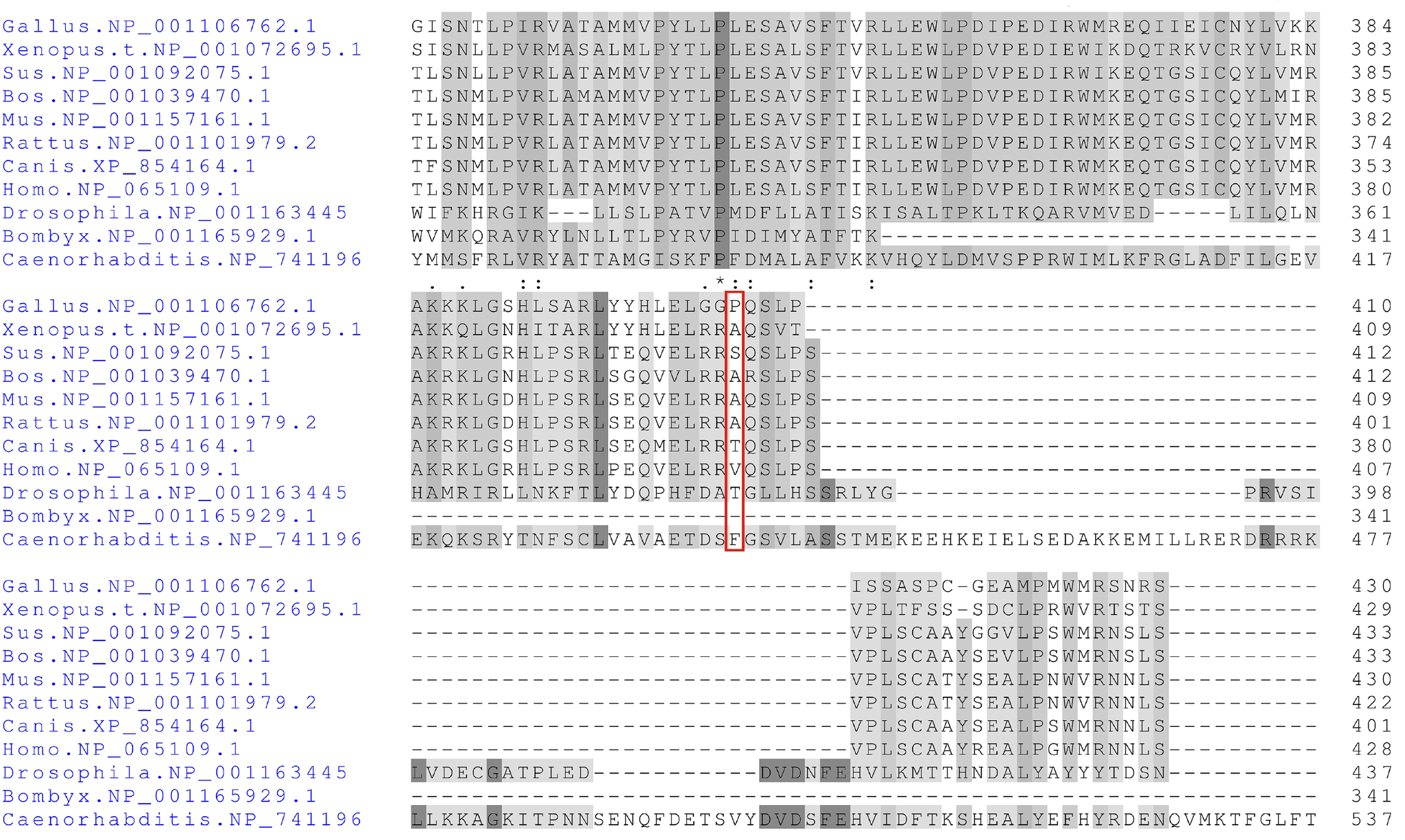

Supplement: Figure S5 — Alignment of the C-terminal region surrounding V402 (screenshot of the ClustalW tool on the Uniprot homepage). The position of V402 is marked by the red rectangle. The protein sequence immediately before V402 is highly conserved in mammals and vertebrates. (1.43 MB TIF) [file pgen.1001239.s005.tif]

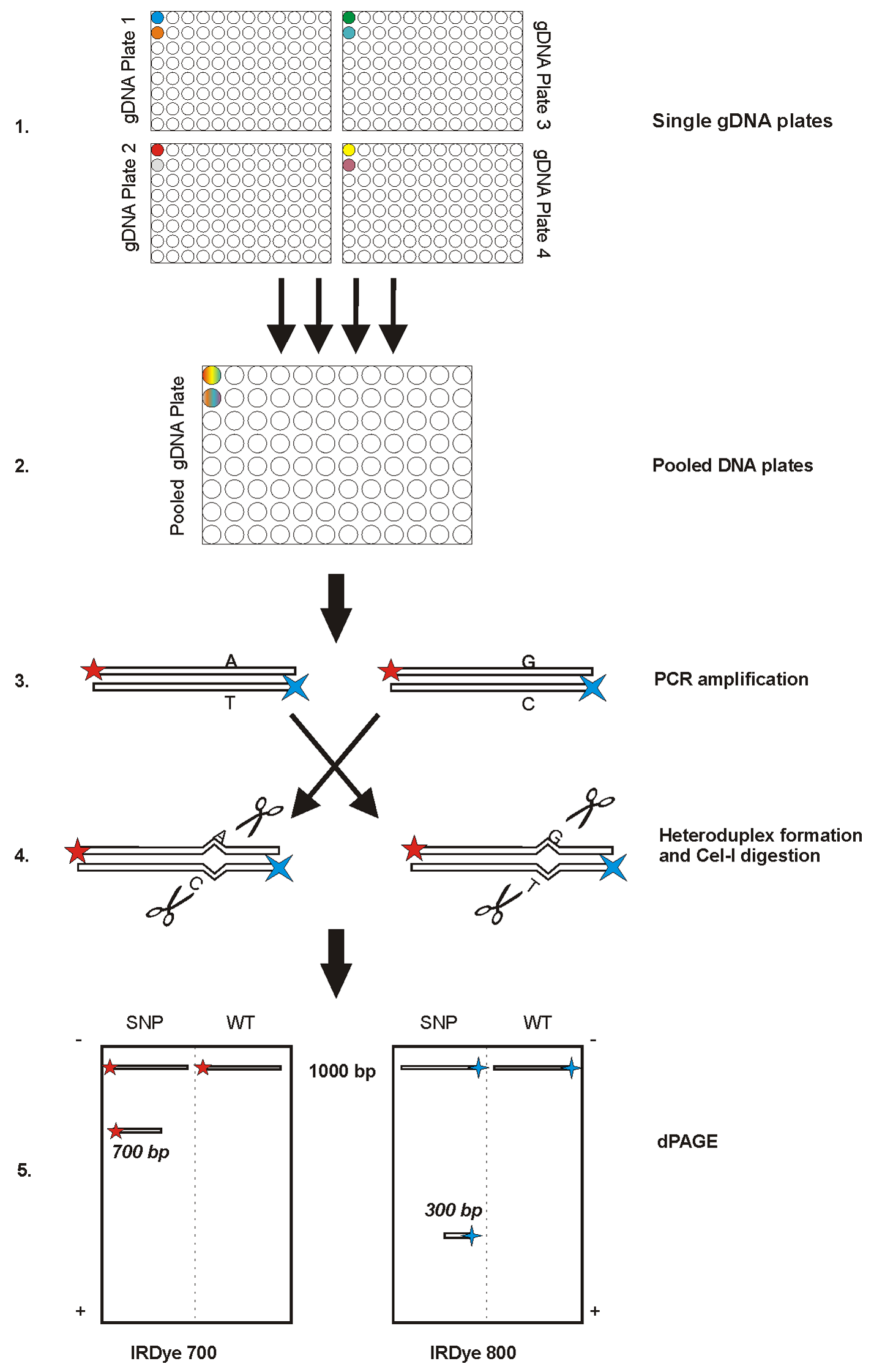

Supplement: Figure S6 — Description of the Ecotilling process. STEP 1: The gDNA is normalized to 20 ng and arrayed on 96 well plates. STEP 2: Every four plates are pooled and normalized to 10 ng. STEP 3: The pooled DNA is used as a template for PCR amplification of the region of interest, employing primers labeled with IRDye 700 for the forward primer (red star), respectively the IRDye 800 for the reverse primer (blue star). STEP 4: The PCR fragments are heat-denatured and slowly renatured in order to allow the formation of heteroduplexes of double-stranded DNA from different individuals. In case that a SNP is present in this fragment, a subpopulation of double-strands will contain a mismatch, which will be cut by the Cel-I endonuclease in the next step. The amplification products are then partially digested with the endonuclease Cel-I which recongises DNA mismatches with high specificity and cuts on their 3′ end. This produces for each mismatch (respectively SNP) two digestion fragments of complementary length and with different labels on their 3′ end. STEP 5: The digestion products are size-fractionized using in a denaturing PAGE and both wavelengths are detected simultaneously. Each SNP produces two additional bands, besides the signal of the undigested full-size product, whose length sums up to the length of the full-size product. Each SNP is doubly detected and therefore quality controlled. (0.52 MB TIF) [file pgen.1001239.s006.tif]

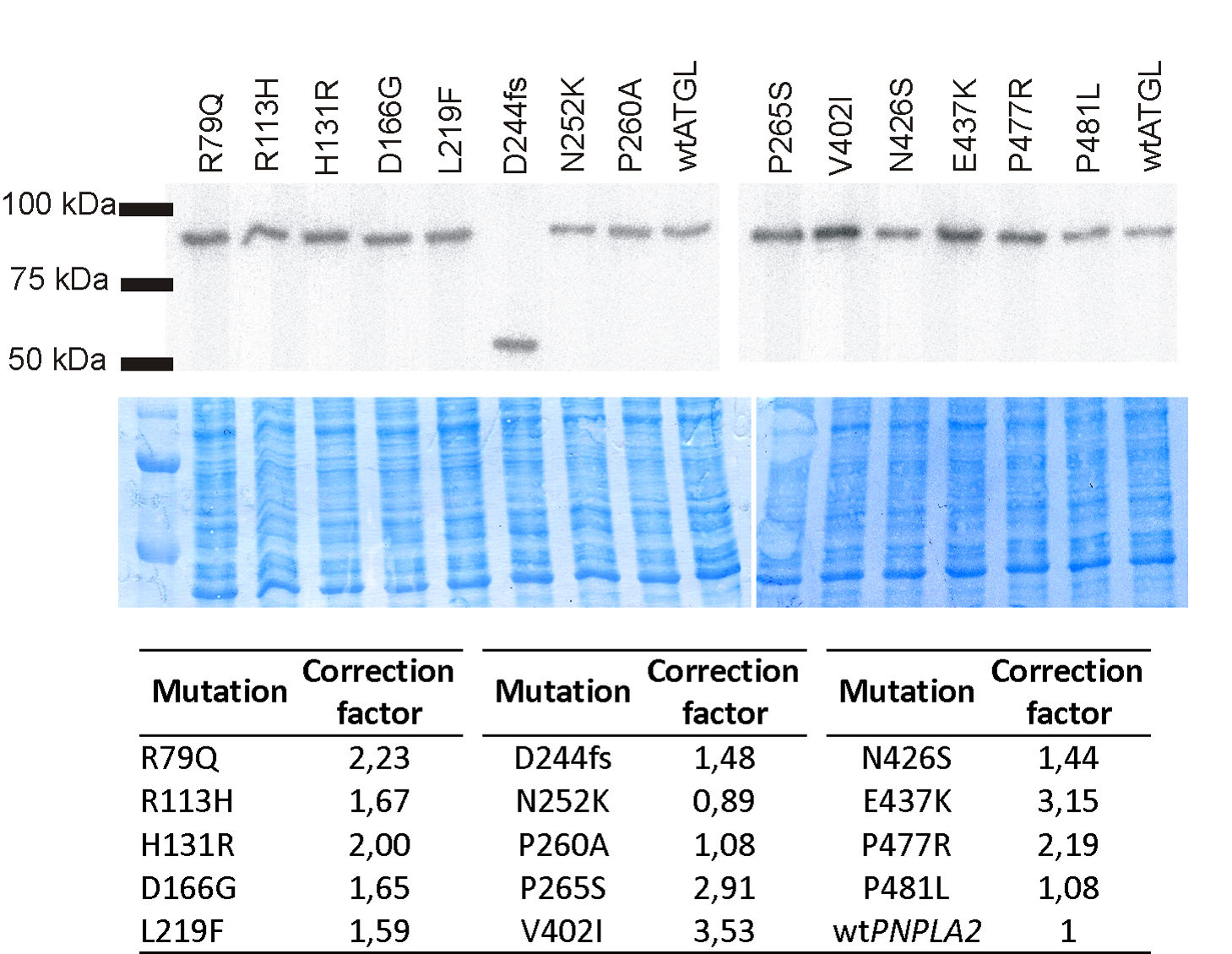

Supplement: Figure S7 — Western blot analysis of the Cos-7 cell lysates used for triglyceride hydrolase assays. Relative expression levels and correction factors for triglyceride hydrolase activities were determined as described in the main text and are given in the table below. Coomassie stained blots are shown for loading control. (1.26 MB TIF) [file pgen.1001239.s007.tif]
