# Supplementary material for: Investigation and Functional Characterization of Rare Genetic Variants in the Adipose Triglyceride Lipase in a Large Healthy Working Population
Source: PLoS Genet. 2010 Dec 9;6(12):e1001239. doi: 10.1371/journal.pgen.1001239 (PMC3000363; doi:10.1371/journal.pgen.1001239)
Supplement: Table S1 — New variations detected in the SAPHIR population. (0.11 MB DOC) [file pgen.1001239.s008.doc]

| **#** | **SNP** | | **Protein** | **Localization** | **n**  **(Aa/aa)** | | **5’ flanking sequence** | | **Wild type  allele** | **Mutated allele** | **3’ flanking sequence** | **MAF [%]** | **NCBI_ss#** | |
| --- | --- | --- | --- | --- | --- | --- | --- | --- | --- | --- | --- | --- | --- | --- |
| 1 | SNP -2266 | |  | upstream | 1/0 | | TGGGATTACAGGTGTGAGCC | | A | C | CTGTGCCTGGCCTATTTTTA | 0.03 | 262967432 | |
| 2 | SNP -2210 | |  | upstream | *na* | | TGGAATCAAGCTATGTTGCC | | T | A | AGGCTGGTCTCAAACTTCTG | > 5.00^a^ | 262967435 | |
| 3 | SNP -2190 | |  | upstream | 5/0 | | TAGGCTGGTCTCAAACTTCT | | G | A | GACTCAAGCAATCCCCCCAC | 0.17 | 262967436 | |
| 4 | SNP -2157 | |  | upstream | *na* | | CCCCCCACCTTGGCCTCCCA | | G | C | AGTGCTGGGATTATAGGTGT | *na* | 262967438 | |
| 5 | SNP -2014 | |  | upstream | *na* | | CTCACTCCCCGACAATCTTC | | G | A | CCTGTCCTGGTCGGCTGCCT | *na* | 262967440 | |
| 6 | SNP -1716 | |  | upstream | 1/0 | | TAGGACTACATGCGTGCACT | | A | G | CCACACCTGGCAAATTTTTG | 0.03 | 262967442 | |
| 7 | SNP -1689 | |  | upstream | 1/0 | | CTGGCAAATTTTTGTATTTT | | C | T | AGTAGAGACAGGGTTTTGCC | 0.03 | 262967444 | |
| 8 | SNP -1678 | |  | upstream | *na* | | TTGTATTTTCAGTAGAGACA | | G | A | GGTTTTGCCATGTTGGCCTG | *na* | 262967446 | |
| 9 | SNP -1596 | |  | upstream | 1/0 | | GGCCTCCCAAAGTGCTGGGA | | T | C | TACAGGCATGGGCCACTGTG | 0.03 | 262967448 | |
| 10 | SNP -1582 | |  | upstream | 1/0 | | CTGGGATTACAGGCATGGGC | | C | T | ACTGTGCCCGGCCTCTTTTT | 0.03 | 262967453 | |
| 11 | SNP -1430 | |  | upstream | 1/0 | | AGCTGGGATTACAGGCGTGT | | G | A | CCACCCTGACCGGCTAATTT | 0.03 | 262967455 | |
| 12 | SNP -1137 | |  | upstream | 1/0 | | GGCCCCATCCTGTTCTGGCC | | C | G | GTTCTGGCTTCCCTAACTCA | 0.03 | 262967457 | |
| 13 | SNP -1097 | |  | upstream | 7/0 | | CAGCTTGGGTTGGGGGCAGC | | C | T | CCAAGCCCCATCCCAGACCC | 0.24 | 262967459 | |
| 14 | SNP -1057 | |  | upstream | 1/0 | | CTCTGCAGCTCTGGCAACAG | | C | T | TAGGTCCAGAGATGACCTCA | 0.03 | 262967460 | |
| 15 | SNP -1039 | |  | upstream | 1/0 | | AGCTAGGTCCAGAGATGACC | | T | G | CAGCCTGGGGAGCCAGTGTT | 0.03 | 262967462 | |
| 16 | SNP -885 | |  | upstream | 1/0 | | CACTGACCAGGAGAAAAATG | | C | T | GGAAAAGGGGTGAAAGGGTG | 0.03 | 262967464 | |
| 17 | SNP -636 | |  | Intron | 1/0 | | GGGACTCGCATCCGGCCTTG | | G | C | CCTTGGATGTTTATGGGTCT | 0.03 | 262967466 | |
| (CONTINUES) | | | | | | | | | | | | | |  |
| (CONTINUES) | | | | | | | | | | | | | |  |
| **#** | **SNP** | | **Protein** | **Localization** | **n**  **(Aa/aa)** | **5’ flanking sequence** | | | **Wild type allele** | **Mutated allele** | **3’ flanking sequence** | **MAF [%]** | **NCBI_ss#** | |
| 18 | SNP -381 | |  | Intron 1 | 2/0 | CGGGCCTGCGCCCAGCCCAC | | | C | T | CTACACCCCTAGGCGTGTGC | 0.07 | 262967467 | |
| 19 | SNP -212 | |  | Intron 1 | 159/3 | GCGCGCCCCGATTGGTCTTC | | | G | A | TGTGCCGGCCCCGCCCCCGC | 4.79 | 262967469 | |
| 20 | Indel+216 | |  | Intron 2 | 1/0 | GCCGGGGGCGGCAGGCGGGG | | | GG | T | CTGGCGGGAAGGCCGTGCGG | 0.03 | 262967530 | |
| 21 | Del +776 | |  | Intron 2 | 1/0 | GCGGCCCACCGCGTTTGCAC | | | AC | - | TTCATGGGTGAGGGTGCTTC | 0.03 | 262967533 | |
| 22 | SNP +987 | |  | Intron 2 | 1/0 | GGCTCACCCCTGCCCTCTTC | | | C | T | TCTGAACTTTGTCCTGGGAG | 0.03 | 262967471 | |
| 23 | SNP +1354 | |  | Intron 2 | 1/0 | TTGGACCTCAGCTCCTCCCT | | | C | T | AGTGCTCCCGACCACTTCCA | 0.03 | 262967473 | |
| 24 | SNP +1565 | |  | Intron 2 | 9/0 | TTGTGGGTCTGCCCCATCCC | | | C | T | GCACTGCTGGATCTGGCCAA | 0.31 | 262967475 | |
| 25 | SNP +1677 | |  | Intron 2 | 1/0 | AGGCCCAGTGACTCATAGGG | | | C | T | AGGCAGTTGGGAAATACCAG | 0.03 | 262967476 | |
| 26 | SNP +1808 | |  | Intron 2 | 1/0 | CACATTCACTGGGCCTCCTC | | | C | T | AGGGTCTGTATGCCATGGAA | 0.03 | 262967478 | |
| 27 | SNP +1958 | | p.R79Q | Exon 3 | 3/0 | ATCTAAAGAGGCCCGGAAGC | | | G | A | GTTCCTGGGCCCCCTGCACC | 0.1 | 262967480 | |
| 28 | SNP +2060 | | p.R113H | Exon 3 | 1/0 | CCATGAGCATGCCAGTGGGC | | | G | A | CCTGGGCATCTCCCTGACCC | 0.03 | 262967482 | |
| 29 | SNP +2091 | | p.D123D | Exon 3 | 1/0 | TCCCTGACCCGCGTGTCAGA | | | C | T | GGCGAGAATGTCATTATATC | 0.03 | 262967484 | |
| 30 | SNP +2114 | | p.H131R | Exon 3 | 1/0 | CGAGAATGTCATTATATCCC | | | A | G | CTTCAACTCCAAGGACGAGC | 0.03 | 262967486 | |
| 31 | SNP +2176 | |  | Intron 3 | 3/0 | GAGCCATGCTGGGTGGCGGT | | | G | C | GGGGGGGCAGTGGGAACCTC | 0.1 | 262967488 | |
| 32 | SNP +2209 | |  | Intron 3 | 5/0 | GGAACCTCAAGGCCTCTGCT | | | C | T | ATTCTCTCCCACTCTGTCCC | 0.17 | 262967490 | |
| 33 | SNP +2410 | |  | Intron 4 | 1/0 | CAGGCCCTTGCTCTGCCACC | | | G | A | CCTGTTACCCACTTCCCCTG | 0.03 | 262967492 | |
| 34 | Del +2591 | |  | Intron 4 | 2/0 | GGTAGCCACTGAATGGGGCC | | | CTTGGT | - | GGCCGGGTGGGGTGGCTGGG | 0.07 | 262967535 | |
| 35 | SNP +2641 | |  | Intron 4 | 1/0 | GGTGGCCAGTGCAGCCACAG | | | G | A | CCCTCACATACGGTCCTGTC | 0.03 | 262967493 | |
| (CONTINUES) | | | | | | | | | | | | | |  |
| (CONTINUED) | | | | | | | | | | | | | |  |
| **#** | **SNP** | **Protein** | | **Localization** | **n (Aa/aa)** | | | **5’ flanking sequence** | **Wildtype allele** | **Mutated allele** | **3’ flanking sequence** | **MAF [%]** | **NCBI_ss#** | |
| 36 | SNP+2847 | p.L219F | | Exon 5 | 1/0 | | | TCCAGTTCAACCTGCGCAAC | C | T | TCTACCGCCTCTCCAAGGCC | 0.03 | 262967495 | |
| 37 | SNP +2961 |  | | Intron 5 | 2/0 | | | CAAGGGAGAACACTGATCCT | T | C | TGACTTCTGAGTGCCCAGGG | 0.07 | 262967497 | |
| 38 | SNP +3086 |  | | Intron 5 | 1/0 | | | GCTGCTCTGTCCAGGCTCCC | T | C | GTCCAGTCTCTCTCTCTTTT | 0.03 | 262967499 | |
| 39 | SNP +3130 |  | | Intron 5 | 1/0 | | | TTTTTTTTTTTGTTTGAGAC | G | A | GAGTCTCGCTCTGTTGCCAA | 0.03 | 262967501 | |
| 40 | Del +3323 |  | | Intron 5 | 2/0 | | | TTTCACCATGTTGGCCAGGC | GGC | - | CTCAAACTATTTTATTTTTT | 0.07 | 262967537 | |
| 41 | SNP +3432 |  | | Intron 5 | 1/0 | | | TCACTGCAAGCTCCACCTCC | C | T | GGGTTCCTGCCATTCTCCTG | 0.03 | 262967503 | |
| 42 | SNP +3449 |  | | Intron 5 | 9/0 | | | TCCCGGGTTCCTGCCATTCT | C | T | CTGCCTCAGCCTCCCCAGTA | 0.31 | 262967505 | |
| 43 | SNP +3838 | p.Y242Y | | Exon 6 | 1/0 | | | GAGATGTGCAAGCAGGGATA | C | T | CGGGATGGCCTGCGCTTTCT | 0.03 | 262967507 | |
| 44 | Del +3844 | p.D244fs | | Exon 6 | 1/0 | | | TGCAAGCAGGGATACCGGGA | T | - | GGCCTGCGCTTTCTGCAGCG | 0.03 | 262967539 | |
| 45 | SNP +3868 | p.N252K | | Exon 6 | 26/0 | | | CTGCGCTTTCTGCAGCGGAA | C | G | GGTGCGCGGACCCGGGCGGG | 0.88 | 262967509 | |
| 46 | SNP +3885 |  | | Intron 6 | 1/0 | | | GAACGGTGCGCGGACCCGGG | C | T | GGGAGAGGGCGGGGTGGGCT | 0.03 | 262967511 | |
| 47 | SNP +3996 | p.P260A | | Exon 7 | 1/0 | | | GCCTCCTGAACCGGCCCAAC | C | G | CCTTGCTGGCGTTGCCCCCC | 0.03 | 262967513 | |
| 48 | SNP +4121 | p.P301P | | Exon 7 | 2/0 | | | CACATCCTGGAGCACCTGCC | C | T | GCCCGGCTCAATGAGGGTGC | 0.07 | 262967515 | |
| 49 | SNP +4141 |  | | Intron 7 | 1/0 | | | CGCCCGGCTCAATGAGGGTG | C | T | CACCTGGGGGACGGGAGGGG | 0.03 | 262967517 | |
| 50 | SNP +4692 | p.R383R | | Exon 9 | *na* | | | CTGGTGATGCGCGCCAAGAG | G | A | AAGCTGGGCAGGCACCTGCC | *na* | 262967519 | |
| 51 | SNP +4833 | p.V402I | | Exon 10 | 1/0 | | | AGCAGGTGGAGCTGCGCCGC | G | A | TCCAGTCGCTGCCGTCCGTG | 0.03 | 262967521 | |
| 52 | SNP +4906 | p.N426S | | Exon 10 | 1/0 | | | GCCCGGCTGGATGCGCAACA | A | G | CCTCTCGCTGGGGGACGCGC | 0.03 | 262967523 | |
| 53 | SNP +4938 | p.E437K | | Exon 10 | 1/0 | | | GGGACGCGCTGGCCAAGTGG | G | A | GGAGTGCCAGCGCCAGCTGC | 0.03 | 262967525 | |
| (CONTINUES) | | | | | | | | | | | | | |  |
| (CONTINUED) | | | | | | | | | | | | | |  |
| **#** | **SNP** | | **Protein** | **Localization** | **n (Aa/aa)** | | **5’ flanking sequence** | | **Wildtype allele** | **Mutated allele** | **3’ flanking sequence** | **MAF [%]** | **NCBI_ss#** | |
| 54 | SNP +5059 | | p.P477R | Exon 10 | 2/0 | | CCCCGCGGACCCAGCATCCC | | C | G | GCAGCACCAGCTGGCCGGGC | 0.07 | 262967527 | |
| 55 | SNP +5432 | |  | 3' UTR | 1/0 | | CCCTCCCCGTTTTTCATGGC | | C | T | TGCTGAAATATGTGTGTGAA | 0.03 | 262967529 | |
