# Supplementary material for: Investigation and Functional Characterization of Rare Genetic Variants in the Adipose Triglyceride Lipase in a Large Healthy Working Population
Source: PLoS Genet. 2010 Dec 9;6(12):e1001239. doi: 10.1371/journal.pgen.1001239 (PMC3000363; doi:10.1371/journal.pgen.1001239)
Supplement: Table S2 — PCR conditions for Ecotilling screening (LI-COR IRDye-labeled). (0.04 MB DOC) [file pgen.1001239.s009.doc]

# Table S2: PCR conditions for Ecotilling screening (LI-COR IRDye-labeled)

| **Fragment** | **bp** | **Labeled Primer Fw**  **(IRDye 700)** | **Labeled**  **Primer Rv**  **(IRDye 800)** | **DNA [ng]** | **dNTP [µM]** | **Fw [µM]** | **Rv [µM]** | **Annealing**  **[°C]** | **Ext. time [sec]** | **Fin.Ext [min]** | **Cycles** | **Touchdown for**  **10 additional cycles** | **DMSO [%]** |
| --- | --- | --- | --- | --- | --- | --- | --- | --- | --- | --- | --- | --- | --- |
| **ATGL 1** | 1214 | ATGL_PCR2_fw | ATGL_PCR3_rv | 60 | 250 | 0.25 | 0.25 | 63 | 40 | 10 | 25 | -0.5°C/cycle | 5 |
| **ATGL 2** | 1125 | ATGL-2_fw | ATGL-2_rv | 15 | 250 | 0.25 | 0.25 | 65 | 40 | 3 | 30 |  |  |
| **ATGL 3** | 1204 | ATGL-3_fw | ATGL_PCR6_rv | 30 | 250 | 0.25 | 0.25 | 65 | 40 | 3 | 30 |  |  |
| **ATGL 4** | 1393 | ATGL-4_fw | ATGL-4_rv | 60 | 250 | 0.25 | 0.25 | 59 | 45 | 10 | 30 | -0.5°C/cycle |  |
| **ATGL 5** | 1168 | ATGL-5_fw | ATGL-5_rv | 15 | 250 | 0.25 | 0.25 | 65 | 40 | 3 | 35 |  |  |
| **ATGL 6** | 1349 | ATGL-6_fw | ATGL-6_rv | 15 | 250 | 0.25 | 0.25 | 65 | 40 | 3 | 35 |  |  |
| **ATGL 7** | 1286 | ATGL-7_fw | ATGL-7_rv | 30 | 250 | 0.25 | 0.25 | 65 | 40 | 3 | 35 |  |  |
| **ATGL 8** | 1511 | ATGL-8_fw | ATGL-8_rv_long2 | 60 | 250 | 0.25 | 0.25 | 65 | 40 | 3 | 35 |  |  |

All PCR reactions were performed in a 15 µl reaction volume using Herculase II Fusion DNA Polymerase (Stratagene, USA).
