# Supplementary material for: Investigation and Functional Characterization of Rare Genetic Variants in the Adipose Triglyceride Lipase in a Large Healthy Working Population
Source: PLoS Genet. 2010 Dec 9;6(12):e1001239. doi: 10.1371/journal.pgen.1001239 (PMC3000363; doi:10.1371/journal.pgen.1001239)
Supplement: Table S4 — Cycling and sequencing protocols for ATGL Ecotilling. (0.04 MB DOC) [file pgen.1001239.s011.doc]

# Table S4: Cycling and sequencing protocols for *ATGL* Ecotilling

| **Step nr** | **Temp [°C]** | **Time** |  | **Step nr** | **Temp [°C]** | **Time** |
| --- | --- | --- | --- | --- | --- | --- |
| 1 | 98 | 4 min |  | 1 | 96 | 2 min |
| 2 | 98 | 10 sec |  | 2 | 96 | 30 sec |
| 3 | *Ta* | 20 sec |  | 3 | 55 | 20 sec |
| 4 | 72 | *Ext. time* |  | 4 | 60 | 60 sec |
| 5 | Repeat step 2-4 | *n cycles* |  | 5 | Repeat step 2-4 | 30 times |
| 6 | 72 | 10 min |  | 6 | 4 °C | hold |
| 7 | 4 | hold |  | 7 |  |  |

Left: Standard PCR cycling protocol without touchdown loop.

Right: Modified cycle sequencing protocol.

The values in italics are given in Supplementary Tables 2 and 3
