# Supplementary material for: Investigation and Functional Characterization of Rare Genetic Variants in the Adipose Triglyceride Lipase in a Large Healthy Working Population
Source: PLoS Genet. 2010 Dec 9;6(12):e1001239. doi: 10.1371/journal.pgen.1001239 (PMC3000363; doi:10.1371/journal.pgen.1001239)
Supplement: Table S5 — Amplification (A) and sequencing primers (S) for the ATGL gene region. (0.16 MB DOC) [file pgen.1001239.s012.doc]

# Table S5: Amplification (A) and sequencing primers (S) for the *ATGL* gene region.

Sequencing primer concentration 3 µM.

| **Primer name** | **Sequence (5' to 3')** | **Type** |  | **Amplification fragment targeted by the sequencing primers ^a^** | | | | | | | |
| --- | --- | --- | --- | --- | --- | --- | --- | --- | --- | --- | --- |
|  |  |  |  | **1** | **2** | **3** | **4** | **5** | **6** | **7** | **8** |
| **PCR2_fw** | GACCTCGTGATCCACTCACC | **A** |  |  |  |  |  |  |  |  |  |
| **PCR3_rv** | CCAAGCTGAGTTAGGGAAGC | **A** |  |  |  |  |  |  |  |  |  |
| **ATGL2_fw** | AGCTAGGACTACATGCGTGCACTA | **A** |  | **x** | **x** |  |  |  |  |  |  |
| **ATGL2_rv** | CAGACCCATAAACATCCAAGGCCA | **A** |  |  |  | **x** |  |  |  |  |  |
| **ATGL3_fw** | AGACAGAATCTCATTGCCTCCCAC | **A** |  |  | **x** |  |  |  |  |  |  |
| **PCR6_rv** | GCTCACCCAGGCAGACCCCG | **A** |  |  |  |  |  |  |  |  |  |
| **ATGL4_fw** | GCCAACGCCACGCACATCTA | **A** |  |  |  |  |  |  |  |  |  |
| **ATGL4_rv** | AGTGTTGGGCTGGAAGTAGTCA | **A** |  |  |  |  |  | **x** |  |  |  |
| **ATGL5_fw** | TGCTCCCAACAAGACTCCTTCTCA | **A** |  |  |  |  | **x** |  |  |  |  |
| **ATGL5_rv** | CTCCTCCTCTGTCCCTCATCCCA | **A** |  |  |  |  |  |  | **x** |  |  |
| **ATGL6_fw** | ATCCCACTTCAACTCCAAGGACGA | **A** |  |  |  |  |  | **x** |  |  |  |
| **ATGL6_rv** | TGAGGCAGGAGAATGGCAGGAA | **A** |  |  |  |  |  |  |  |  |  |
| **ATGL7_fw** | AACCTCTCTGTCTAGCTGCTCTGT | **A** |  |  |  |  |  |  |  | **x** |  |
| **ATGL7_rv** | AGCATGTTGGAGAGGGTGGTCAG | **A** |  |  |  |  |  |  |  |  | **x** |
| **ATGL8_fw** | AGAAGATCACATCCTGGAGCACCT | **A** |  |  |  |  |  |  |  | **x** |  |
| **ATGL8_rv_long2** | TGCAGCTGTGGAGAGGCT | **A** |  |  |  |  |  |  |  |  | **x** |
| **ATGL1_fw** | AGGCTGGTCTCAAACTTCTGGACT | **S** |  | **x** |  |  |  |  |  |  |  |
| **ATGL1_rv** | AAGCTCACACCTGTAATCCCAGCA | **S** |  | **x** | **x** |  |  |  |  |  |  |
| **ATGL1_Seq_fw1** | AGAAGAAGTCATGCTCTGTCACCTGG | **S** |  | **x** |  |  |  |  |  |  |  |
| **ATGL1_Seq_rv1** | CCATGCCTGTAATCCCAGCACTTT | **S** |  | **x** | **x** |  |  |  |  |  |  |
| **ATGL1_Seq_rv2** | AGTGATGCTGAGTTGAATTT | **S** |  | **x** |  |  |  |  |  |  |  |
| **ATGL2_Seq_fw1** | AAACTCCCGACTTTCTGTGATCCG | **S** |  | **x** | **x** |  |  |  |  |  |  |
| **ATGL2_Seq_rv1** | GTGGGAGGCAATGAGATTCTGTCT | **S** |  |  | **x** |  |  |  |  |  |  |
| **ATGL3_rv** | ATTGGATCCTCGCCGGGTT | **S** |  |  |  |  | **x** |  |  |  |  |
| **ATGL3_for1** | CTCCCACCCCAACCTGAG | **S** |  |  |  | **x** |  |  |  |  |  |
| **ATGL3_for2** | GCCCAGCCCACCCTACAC | **S** |  |  |  | **x** |  |  |  |  |  |
| **ATGL3_for3** | CTTGGATGTTTATGGGTC | **S** |  |  |  | **x** |  |  |  |  |  |
| **ATGL3_for4** | CGGCTCCAGCGAGCGAG | **S** |  |  |  | **x** |  |  |  |  |  |
| **ATGL3_rev1** | CAGCGCCGTGGCCGTGAG | **S** |  |  |  | **x** |  |  |  |  |  |
| **ATGL3_rev2** | GACCTGGGTACCGGCGAG | **S** |  |  |  | **x** |  |  |  |  |  |
| **ATGL3_rev3** | CGGCCAGGCCTCTGTGAG | **S** |  |  |  | **x** |  |  |  |  |  |
| **ATGL3_rev4** | AGACCCACGCGACCTGAC | **S** |  |  |  | **x** |  |  |  |  |  |
| (continues) | | | | | | | | | | | |
|  | | | | | | | | | | | |
|  | | | | | | | | | | | |
|  | | | | | | | | | | | |
| (continued) | | | | | | | | | | | |
| **Primer name** | **Sequence (5' to 3')** | **Type** |  | **Amplification fragment targeted by the sequencing primers ^a^** | | | | | | | |
|  |  |  |  | **1** | **2** | **3** | **4** | **5** | **6** | **7** | **8** |
| **ATGL3_rev5** | GGCACACGAAGACCAATC | **S** |  |  |  | **x** |  |  |  |  |  |
| **ATGL3_Seq_rv1** | CGTAGATGTGCGTGGCGTT | **S** |  |  |  | **x** |  |  |  |  |  |
| **ATGL3_Seq_rv2** | AGAAAGCTTCTGCCCTCCCT | **S** |  |  |  | **x** |  |  |  |  |  |
| **ATGL3_Seq_rv3** | CCAGACCTGAAAGCCGCACA | **S** |  |  |  | **x** |  |  |  |  |  |
| **ATGL4_for1** | GCACATCTACGGCGCCTC | **S** |  |  |  |  | **x** |  |  |  |  |
| **ATGL4_for2** | TGCACACGGTGCCCTGTG | **S** |  |  |  |  | **x** |  |  |  |  |
| **ATGL4_rev1** | GTAGTCAGGGCCCAGTTC | **S** |  |  |  |  | **x** |  |  |  |  |
| **ATGL4_rev2** | GTGCTGTAAACATCACTC | **S** |  |  |  |  | **x** |  |  |  |  |
| **ATGL4_rev3** | AGCGACCCGGATTGGATC | **S** |  |  |  |  | **x** |  |  |  |  |
| **ATGL4_Seq_fw1** | TGGATATTACAACCGGTGCCAGC | **S** |  |  |  |  | **x** |  |  |  |  |
| **ATGL4_Seq_fw2** | TTTACAGCACACGACTTCAGGAGC | **S** |  |  |  |  | **x** |  |  |  |  |
| **ATGL4_Seq_rv1** | TGTGCAAGGAGAGGGCAGGAGA | **S** |  |  |  |  | **x** |  |  |  |  |
| **ATGL5_Seq_fw1** | TCATAGGGCAGGCAGTTGGGAAATAC | **S** |  |  |  |  |  | **x** |  |  |  |
| **ATGL5_Seq_fw3** | CCCTCCTTCAACCTGGTAAA | **S** |  |  |  |  |  | **x** |  |  |  |
| **ATGL5_Seq_fw4** | GACATGGGGCTATGAAGGAA | **S** |  |  |  |  |  | **x** |  |  |  |
| **ATGL5_Seq_rv1** | TCGTCCTTGGAGTTGAAGTGGGAT | **S** |  |  |  |  |  | **x** |  |  |  |
| **ATGL5_Seq_rv2** | ATGTCCAGGGTTCCATGGCATACA | **S** |  |  |  |  |  | **x** |  |  |  |
| **ATGL6_Seq_fw2** | GACAACCTGCCACTCTATGAGCTT | **S** |  |  |  |  |  |  | **x** |  |  |
| **ATGL6_Seq_fw4** | CACCAAGGGAGAACACTGATCCTT | **S** |  |  |  |  |  |  | **x** |  |  |
| **ATGL6_Seq_fw5** | TTCAAGCTATTCTCCTGCCTCAGC | **S** |  |  |  |  |  |  | **x** |  |  |
| **ATGL6_Seq_fw6** | TCCTTCAGGCCCTTGCTCTG | **S** |  |  |  |  |  |  | **x** |  |  |
| **ATGL6_Seq_fw7** | ACTGATCCTTTGACTTCTGA | **S** |  |  |  |  |  |  | **x** |  |  |
| **ATGL6_Seq_rv1** | GTTGAACTGGATGCTGGTGTTGGT | **S** |  |  |  |  |  |  | **x** |  |  |
| **ATGL6_Seq_rv2** | ACAGAGCAGCTAGACAGAGAGGTT | **S** |  |  |  |  |  |  | **x** |  |  |
| **ATGL6_Seq_rv3 ^b^** | TAGTTTGAGGCCAGCCTG | **S** |  |  |  |  |  |  | **x** |  |  |
| **ATGL6_Seq_rv4** | CAGCCTGGCCAACATGGT | **S** |  |  |  |  |  |  | **x** |  |  |
| **ATGL7_Seq_fw1** | ATGAAGTCTCACACTGTCACCCAG | **S** |  |  |  |  |  |  |  | **x** |  |
| **ATGL7_Seq_fw2** | TCCAACCTCCCTGTACAGCTTTGA | **S** |  |  |  |  |  |  |  | **x** |  |
| **ATGL7_Seq_fw3** | GTCTTGATCTCCTGACCTCGTCAT | **S** |  |  |  |  |  |  |  | **x** |  |
| **ATGL7_Seq_fw5** | TAGTGAAGGGAGGTGGCTGTT | **S** |  |  |  |  |  |  |  | **x** |  |
| **ATGL7_Seq_fw7** | AGAGGACAAGGACCAGGCAGT | **S** |  |  |  |  |  |  |  | **x** |  |
| **ATGL7_Seq_rv1** | AGGGATTCTCAGTTCATCAGCG | **S** |  |  |  |  |  |  |  | **x** |  |
| **ATGL7_Seq_rv2** | CAAAACCTATCCTAGATCCA | **S** |  |  |  |  |  |  |  | **x** |  |
| **ATGL7_Seq_rv3** | GGTTGGACAGAAAGC | **S** |  |  |  |  |  |  |  | **x** |  |
| **ATGL8_rv** | TCACACACATATTTCAGCAGGCCA | **S** |  |  |  |  |  |  |  |  | **x** |
| **ATGL8_Seq_fw4** | ACCTGTGCCTTAATCTTCCCTCCC | **S** |  |  |  |  |  |  |  |  | **x** |
| (continues) | | | | | | | | | | | |
|  | | | | | | | | | | | |
| (continued) | | | | | | | | | | | |
| **Primer name** | **Sequence (5' to 3')** | **Type** |  | **Amplification fragment targeted by the sequencing primers ^a^** | | | | | | | |
|  |  |  |  | **1** | **2** | **3** | **4** | **5** | **6** | **7** | **8** |
| **ATGL8_Seq_fw7** | CCAAGTGGGAGGAGTGCCAG | **S** |  |  |  |  |  |  |  |  | **x** |
| **ATGL8_Seq_rv1** | TTGGTGCAGAAGAGGCCGA | **S** |  |  |  |  |  |  |  |  | **x** |
| **ATGL8_Seq_rv2** | TCTTGGCGCGCATCACCAGGTACT | **S** |  |  |  |  |  |  |  |  | **x** |
| **ATGL8_Seq_rv3** | CCCTCTTGGCAACTGTGAGTCC | **S** |  |  |  |  |  |  |  |  | **x** |
| **ATGL8_Seq_rv4** | TAATGGAGGCGTCTCAGGCAGGGTT | **S** |  |  |  |  |  |  |  |  | **x** |
| **ATGL8_rv_long** | TAAGCAGGCGGTCACATACACA | **S** |  |  |  |  |  |  |  |  | **x** |

^a^ Due to the overlapping PCR fragments, some amplification primers serve also as sequencing primer. ^b^ Needs 2.5% DMSO in the cycle sequencing reaction.
