# Supplementary material for: Investigation and Functional Characterization of Rare Genetic Variants in the Adipose Triglyceride Lipase in a Large Healthy Working Population
Source: PLoS Genet. 2010 Dec 9;6(12):e1001239. doi: 10.1371/journal.pgen.1001239 (PMC3000363; doi:10.1371/journal.pgen.1001239)
Supplement: Table S6 — PCR conditions for site directed mutagenesis. (0.05 MB DOC) [file pgen.1001239.s013.doc]

# Table S6: PCR conditions for site directed mutagenesis.

| **Mutant** | **Primer Fw** | **Primer Rv** | **Methylated Plasmid [ng]** | **dNTP [µM]** | **Fw [µM]** | **Rv [µM]** | **Annealing**  **[°C]** | **Cycles** | **DMSO**  **[%]** |
| --- | --- | --- | --- | --- | --- | --- | --- | --- | --- |
| **R79Q** | ATGL-R79Q_fw | ATGL-R79Q_rv | 12.5 | 200 | 0. 5 | 0. 5 | 65 | 30 | 1,5 |
| **R113H** | ATGL-R113H_fw | ATGL-R113H_rv | 12.5 | 200 | 0. 5 | 0. 5 | 65 | 30 | 1,5 |
| **H131R** | ATGL-H131R_fw | ATGL-H131R_rv | 12.5 | 200 | 0. 5 | 0. 5 | 65 | 30 | 1,5 |
| **D166G** | ATGL-D166G_fw | ATGL-D166G_rv | 12.5 | 200 | 0. 5 | 0. 5 | 65 | 30 | 1,5 |
| **L219F** | ATGL-L219F_fw | ATGL-L219F_rv | 12.5 | 200 | 0. 5 | 0. 5 | 65 | 30 | 1,5 |
| **D244*fs*** | ATGL-D244fs_fw | ATGL-D244fs_rv | 12.5 | 200 | 0. 5 | 0. 5 | 65 | 30 | 1,5 |
| **N252K** | ATGL-N252K_fw | ATGL-N252K_rv | 12.5 | 200 | 0. 5 | 0. 5 | 65 | 30 | 1,5 |
| **P260A** | ATGL-P260A_fw | ATGL-P260A_rv | 18.75 | 200 | 0. 5 | 0. 5 | 67 | 31 | 5,0 |
| **P265S** | ATGL-P265S_fw | ATGL-P265S_rv | 12.5 | 200 | 0. 5 | 0. 5 | 65 | 30 | 1,5 |
| **V402I** | ATGL-V402I_fw | ATGL-V402I_rv | 18.75 | 200 | 0. 5 | 0. 5 | 67 | 31 | 5,0 |
| **N426S** | ATGL-N426S_fw | ATGL-N426S_rv | 12.5 | 200 | 0. 5 | 0. 5 | 65 | 30 | 1,5 |
| **E437K** | ATGL-E437K_fw | ATGL-E437K_rv | 18.75 | 200 | 0. 5 | 0. 5 | 67 | 31 | 5,0 |
| **P477R** | ATGL-P477R_fw | ATGL-P477R_rv | 18.75 | 200 | 0. 5 | 0. 5 | 67 | 31 | 5,0 |
| **P481L** | ATGL-P481L_fw | ATGL-L481P_rv | 18.75 | 200 | 0. 5 | 0. 5 | 65 | 33 | 1,5 |

PCR Amplification was done using the Phusion High Fidelity DNA Polymerase with GC-rich buffer (Finnzymes, Finland).
