# Supplementary material for: Investigation and Functional Characterization of Rare Genetic Variants in the Adipose Triglyceride Lipase in a Large Healthy Working Population
Source: PLoS Genet. 2010 Dec 9;6(12):e1001239. doi: 10.1371/journal.pgen.1001239 (PMC3000363; doi:10.1371/journal.pgen.1001239)
Supplement: Table S7 — Cycling conditions for site directed mutagenesis. (0.03 MB DOC) [file pgen.1001239.s014.doc]

# Table S7: Cycling conditions for site directed mutagenesis.

| **Step nr** | **Temp** | **Time** |
| --- | --- | --- |
| 1 | 98 °C | 30 sec |
| 2 | 98 °C | 10 sec |
| 3 | *Ta* | 30 sec |
| 4 | 72 °C | 3 min |
| 5 | Goto step 2 | *n cycles* |
| 6 | 72 °C | 10 min |
| 7 | 4 °C | hold |

The values in italics are given in Table S6.
