# Supplementary material for: Investigation and Functional Characterization of Rare Genetic Variants in the Adipose Triglyceride Lipase in a Large Healthy Working Population
Source: PLoS Genet. 2010 Dec 9;6(12):e1001239. doi: 10.1371/journal.pgen.1001239 (PMC3000363; doi:10.1371/journal.pgen.1001239)
Supplement: Table S8 — Primer sequences for site directed mutagenesis. (0.04 MB DOC) [file pgen.1001239.s015.doc]

# Table S8: Primer sequences for site directed mutagenesis

| **#** | **Primer** | **Sequence (5' to 3')** |  | **#** | **Primer** | **Sequence** |
| --- | --- | --- | --- | --- | --- | --- |
| **1** | ATGL-R79Q_fw | ATCTAAAGAGGCCCGGAAGCAGTTCCTGGGC |  | **8** | ATGL-P260A_fw | GCCTCCTGAACCGGCCCAACGCCTTGCTGGC |
|  | ATGL-R79Q_rv | GCTTCCGGGCCTCTTTAGATACCTCAATGAACTTG |  |  | ATGL-P260A_rv | GTTGGGCCGGTTCAGGAGGCCGTTCCGCTGC |
| **2** | ATGL-R113H_fw | ATGAGCATGCCAGTGGGCACCTGGGCATCTCC |  | **9** | ATGL-P265S_fw | AACCCCTTGCTGGCGTTGTCCCCCGCCCGCCCC |
|  | ATGL-R113H_rv | GCCCACTGGCATGCTCATGGCTATCAGCAG |  |  | ATGL-P265S_rv | CAACGCCAGCAAGGGGTTGGGCCGGTTCAGGAGGC |
| **3** | ATGL-H131R_fw | GAGAATGTCATTATATCCCGCTTCAACTCCAAGG |  | **10** | ATGL-V402I_fw | AGCAGGTGGAGCTGCGCCGCATCCAGTCGCT |
|  | ATGL-H131R_rv | GGGATATAATGACATTCTCGCCGTCTGACAC |  |  | ATGL-V402I_rv | GCGGCGCAGCTCCACCTGCTCCGGCAGCCT |
| **4** | ATGL-D166G_fw | CAGGGGGTGCGCTACGTGGGTGGTGGCATTTC |  | **11** | ATGL-N426S_fw | GGCTGGATGCGCAACAGCCTCTCGCTGGGGG |
|  | ATGL-D166G_rv | CCACGTAGCGCACCCCCTGGAGGGAGGGA |  |  | ATGL-N426S_rv | TGTTGCGCATCCAGCCGGGCAGTGCCTCTCTG |
| **5** | ATGL-L219F_fw | CAGTTCAACCTGCGCAACTTCTACCGCCTCTC |  | **12** | ATGL-E437K_fw | GGACGCGCTGGCCAAGTGGAAGGAGTGCCAG |
|  | ATGL-L219F_rv | GTTGCGCAGGTTGAACTGGATGCTGGTGTTG |  |  | ATGL-E437K_rv | CCACTTGGCCAGCGCGTCCCCCAGCGAGA |
| **6** | ATGL-D244fs_fw | AAGCAGGGATACCGGGAGGCCTGCGCTTTC |  | **13** | ATGL-P477R_fw | CCGCGGACCCAGCATCCCGGCAGCACCAGC |
|  | ATGL-D244fs_rv | TCCCGGTATCCCTGCTTGCACATCTCTCGC |  |  | ATGL-P477R_rv | GGGATGCTGGGTCCGCGGGGGCGGGAGCC |
| **7** | ATGL-N252K_fw | TGCGCTTTCTGCAGCGGAAGGGCCTCCTGAACC |  | **14** | ATGL-P481L_fw | ATCCCCGCAGCACCAGCTGGCCGGGCCTG |
|  | ATGL-N252K_rv | TTCCGCTGCAGAAAGCGCAGGCCATCCCG |  |  | ATGL-P481L_rv | GCTGGTGCTGCGGGGATGCTGGGTCCGCG |
