# Supplementary material for: Investigation and Functional Characterization of Rare Genetic Variants in the Adipose Triglyceride Lipase in a Large Healthy Working Population
Source: PLoS Genet. 2010 Dec 9;6(12):e1001239. doi: 10.1371/journal.pgen.1001239 (PMC3000363; doi:10.1371/journal.pgen.1001239)
Supplement: Table S9 — GenBank Accession numbers of the proteins used for conservation analysis. (0.04 MB DOC) [file pgen.1001239.s016.doc]

# Table S9: GenBank accession numbers of the proteins used for conservation analysis

| **Organism** | **GenBank Accession Number** |
| --- | --- |
| Homo sapiens | NP_065109.1 |
| Mus musculus | NP_001157161.1 |
| Rattus norvegicus | NP_001101979.2 |
| Canis familiaris | XP_854164.1 |
| Sus scrofa | NP_001092075.1 |
| Bos taurus | NP_001039470.1 |
| Gallus gallus | NP_001106762.1 |
| Xenopus tropicalis | NP_001072695.1 |
| Drosophila melanogaster | NP_001163445.1 |
| Bombyx mori | NP_001165929.1 |
| Caenorhabditis elegans | NP_741196.1 |
